# Pangenome Dynamics and Functional Diversification in the Marine Genus *Pseudoalteromonas*: Association to Colony Pigmentation

**DOI:** 10.1007/s10126-026-10674-7

**Published:** 2026-07-20

**Authors:** Jéssica Scherer, Renato Kulakowski Corá, Diego Bonatto, Alexandre José Macedo

**Affiliations:** 1 Laboratório de Biofilmes e Diversidade Microbiana, Faculdade de Farmácia e Centro de Biotecnologia, Universidade Federal do Rio Grande do Sul, Porto Alegre, 91501-970 Rio Grande do Sul Brazil; 2 Regenera Moléculas do Mar, Avenida Ipiranga 6681, prédio 96D, sala 210, Partenon, Porto Alegre, RS CEP 90160-091 Brazil; 3 Laboratório de Biologia Molecular e Computacional, Centro de Biotecnologia da UFRGS, Departamento de Biologia Molecular e Biotecnologia, Universidade Federal do Rio Grande do Sul, Porto Alegre, RS Brazil

**Keywords:** *Pseudoalteromonas*, BGCs, Marine bacteria, Comparative genomic, Genome mining

## Abstract

*Pseudoalteromonas* species are ecologically versatile marine bacteria widely recognized for their capacity to synthesize diverse bioactive metabolites and psychrophilic enzymes with biotechnological relevance. Here, we present a comprehensive comparative genomic analysis of 53 reference genomes to elucidate the dynamics, functional diversity, and biosynthetic potential of this genus. Reference genomes representing each deposited species were selected in order to avoid bias associated with unequal numbers of genomes per species. Pangenome reconstruction revealed an open structure comprising a small core genome (1,350 gene families) and a large proportion of accessory and strain-specific genes, reflecting extensive genomic plasticity. Functional annotation indicated that accessory regions are enriched for genes involved in secondary metabolism, stress adaptation, and environmental resilience. Notably, biosynthetic gene cluster (BGC) mining uncovered a rich repertoire of potentially novel RiPPs and other secondary metabolite clusters, underscoring *Pseudoalteromonas* as a promising source of unexplored bioactive compounds. Statistical analyses revealed that pigmented strains harbor significantly higher numbers of BGCs compared to non-pigmented strains, while only a weak and non-significant correlation was observed between BGC abundance and carbohydrate-active enzyme (CAZyme) content. No significant effect of the isolation source was detected on either BGC or CAZyme distributions. Together, these findings provide new insights into the genomic basis of ecological adaptation and metabolic diversification in *Pseudoalteromonas*, supporting the role of pigmentation as a proxy for enhanced biosynthetic potential, while carbohydrate utilization capabilities evolve more independently and offering a framework for targeted bioprospecting of marine-derived metabolites with industrial and environmental applications.

**Supplementary Information:**

The online version contains supplementary material available at 10.1007/s10126-026-10674-7.

## Background

*Pseudoalteromonas* is a genus of Gram-negative marine bacteria, classified as aerobic, non-spore-forming (Gauthier et al. 1995). Members of *Pseudoalteromonas* are widely distributed across marine environments, including seawater, marine sediments, deep-sea habitats, and Polar Regions, reflecting their remarkable ecological versatility. These bacteria are considered cosmopolitan and can represent up to ~ 2.5% of planktonic microbial communities in the oceans and as much as 8% of bacterial diversity in deep-sea environments (~ 4,000 m depth), as well as being dominant in polar ecosystems (Wietz et al. 2010; Wei et al. 2021; Hwang et al. 2023). Their ability to colonize extreme environments, including deep-sea and low-temperature habitats, as well as to establish symbiotic or parasitic associations with a wide range of marine eukaryotes, further highlights their ecological plasticity (Zheng et al. 2023; Hwang et al. 2023). In addition to their ecological relevance, *Pseudoalteromonas* species are widely recognized for their metabolic versatility and their ability to produce diverse bioactive compounds and enzymes with significant biotechnological potential (Offret et al. 2016; Borchert et al. 2017).

The genus currently comprises 70 described species in List of Prokaryotic names with Standing in Nomenclature (LPSN), of which 53 have reference genomes available in public databases such as NCBI. This growing genomic availability has enabled comparative and pangenomic analyses, revealing an open pangenome and substantial genetic diversity within *Pseudoalteromonas*, largely driven by accessory and unique genes (Bosi et al. 2017). However, despite these advances, extending pangenome analyses beyond the species level remains methodologically challenging. In particular, standard sequence similarity thresholds may be overly stringent, leading to an underestimation of core gene sets, whereas more permissive thresholds may increase false-positive clustering. Simply scaling established bioinformatics workflows is insufficient to capture the complexity of increasingly large and diverse genomic datasets. Therefore, novel computational approaches and refined analytical frameworks are required to improve core genome inference at broader taxonomic levels (Lamkiewicz et al. 2024). In this context, platforms such as Anvi’o provide a flexible and integrative framework to mitigate these limitations and enable more robust genus-level pangenomic analyses (Eren et al. 2021).

Building on this genomic framework, the core genome of *Pseudoalteromonas* can be interpreted as a conserved functional backbone that supports survival across diverse marine environments. These conserved genes are primarily associated with essential cellular processes, including central metabolism, cellular maintenance, and stress response, which are critical for persistence under common oceanic conditions such as low temperature, high salinity, and variable nutrient availability, as previously observed in genomic and ecological studies of *Pseudoalteromonas* (Parrilli et al. 2019; Bosi et al. 2017). While the core genome underpins broad environmental adaptability, the accessory genome plays a major role in ecological specialization and niche differentiation (Sonnenberg and Haugen 2021, 2023). In particular, genes associated with carbohydrate-active enzymes (CAZymes) and biosynthetic gene clusters (BGCs) are often enriched in the accessory fraction, enabling the degradation of complex marine polysaccharides derived from algal biomass and particulate organic matter, as well as the production of specialized metabolites involved in ecological interactions such as competition, signaling, and symbiosis. Notably, a substantial proportion of predicted BGCs remains poorly characterized, highlighting a vast and largely unexplored reservoir of metabolic potential (Oliveira et al. 2020; Offret et al. 2016; Navarro-Muñoz et al. 2020; Chau et al. 2021).

Despite these advances, the relationship between genomic diversity and phenotypic traits in *Pseudoalteromonas* remains incompletely understood. In particular, pigmentation, commonly used to differentiate strains, has been associated with the production of bioactive compounds and ecological interactions, yet its functional and evolutionary significance is still not fully resolved (Bowman 2007; Offret et al. 2016). More broadly, there is a lack of integrative studies linking pangenome structure to metabolic capabilities and ecological roles. While pangenomic approaches have provided valuable insights into gene distribution patterns, the extent to which core and accessory gene fractions translate into functional traits, such as metabolite production and environmental adaptation, remains unclear (Li 2025; Fang and Edwards 2025). Bridging this gap is essential to better understand how genomic variation shapes ecological functions and biotechnological potential.

We hypothesized that genomic variation within *Pseudoalteromonas*, particularly within the accessory genome, is associated with phenotypic and ecological traits. Specifically, we tested whether pigmentation and environmental origin (isolation site and oceanic region) correlate with the distribution of BGCs and CAZymes, two functional classes that are central to microbial ecological interactions, resource utilization, and production of bioactive compounds in marine environments. Despite being widely studied separately, these genomic features have rarely been jointly analyzed in an integrated ecological framework within the genus. Additionally, pigmentation has been frequently proposed as a proxy for stress response and ecological adaptation, yet its relationship with broader metabolic potential remains poorly resolved. Furthermore, we hypothesized that while the core genome maintains conserved functions essential for survival across marine environments, the accessory and singleton fractions drive functional diversification and ecological specialization.

In this study, we performed a comprehensive pangenome analysis of the genus *Pseudoalteromonas* using the Anvi’o platform to resolve core and accessory genomic fractions and to investigate singletons as lineage-specific signatures of functional diversification across 53 genomes. By integrating pangenomic structure with pigmentation data and the annotation of BGCs, predicted using antiSMASH and organized with BiG-SCAPE, as well as CAZymes, we investigated how metabolic potential relates to phenotypic traits and ecological origins. This integrative framework provides insights into the extensive biosynthetic novelty and functional plasticity of this marine genus.

## Materials and Methods

### Source *Pseudoalteromonas* Genomes

The 53 genomes used for the pangenome analysis were obtained from the NCBI Reference Sequence Database (RefSeq) and analyzed using the NCBI Datasets tool, yielding a representative set of species described within the genus *Pseudoalteromonas* (accessed in August 2025). Only genomes deposited as reference assemblies were included, ensuring broad coverage and avoiding redundancy from multiple sequencing of the same strain. Completeness and contamination values were derived from RefSeq records, and genomes with > 86% completeness and < 7% contamination were retained for analysis. The absence of cutoff values was selected to ensure full representativeness among the species with available genomes. Genome size, GC content, and coding sequence counts were extracted from the corresponding RefSeq annotations.

### Pangenomics Analysis

All genomes were analyzed using the Anvi’o workflow, an open-source, community-driven platform for microbial-omics (https://anvio.org/*)* (Eren et al. 2021). Anvi’o tools were used to annotate the genomes using information from Clusters of Orthologous Groups (COG), Carbohydrate-Active enZYmes (CAZy), KEGG, and Pfam databases. The pangenome was computed with a minimum bit score of 0.5, an MCL inflation parameter of 10, and inclusion of gene clusters with at least one occurrence in any genome. Orthologous gene clusters were classified as core (present in all genomes), accessory (present in some genomes), or singleton (present in only one genome). Genomic similarity was determined using Average Nucleotide Identity (ANI), computed with the FastANI method, which is also implemented in the Anvi’o tools (Eren et al. 2021).

### Secondary Metabolism Pathway Prediction

To predict BGCs, all 53 genomes were analyzed with antiSMASH 8.0.2 (Blin et al. 2025). Clusters with similarity above 50% to entries in the MIBiG database were considered associated with a known secondary metabolite pathway. In contrast, those below this threshold were classified as putative (orphan gene cluster). To assess the biosynthetic diversity among *Pseudoalteromonas* genomes, a similarity network of BGCs was constructed using BiG-SCAPE v2.0 (Navarro-Muñoz et al. 2020), and the resulting network was visualized with Cytoscape v3.10.3 (Shannon et al. 2003).

### CAZyme Gene Identification

Genes encoding CAZymes - www.cazy.org were identified using dbCAN2 version 5.1.2, a standalone tool of the dbCAN3 online server (Zheng et al. 2023). Protein sequences from all genomes were submitted for annotation. Predicted CAZyme families that synthesize, modify, or break down saccharides were classified according to the CAZy database categories, including glycoside hydrolases (GHs), glycosyltransferases (GTs), polysaccharide lyases (PLs), carbohydrate esterases (CEs), auxiliary activities (AAs), and carbohydrate-binding modules (CBMs). Only hits supported by at least two prediction tools within dbCAN2 were considered for further analyses.

### Phylogenomic Analysis

Phylogenomic relationships within the genus *Pseudoalteromonas* were reconstructed using 53 genomes retrieved from the NCBI RefSeq database. The analysis was performed using the EasyCGTree pipeline (Zhang et al. 2023) with default parameters. To identify core orthologous groups, the bac120 HMM set, comprising 120 ubiquitous bacterial marker genes, was employed. The phylogenomic tree was inferred via the supermatrix approach, and tree reconstruction was executed using FastTree (Price et al. 2009). Finally, the resulting phylogeny was visualized and annotated using the Interactive Tree Of Life (iTOL) v6 (Letunic and Bork 2024).

### Statistical Analysis

Statistical analyses were performed in R (version 4.5.2) using RStudio (2025.9.2.418). Associations between the total number of BGCs predicted by antiSMASH and the total content of CAZymes were evaluated using Spearman’s rank correlation coefficient. Differences in BGC abundance and CAZyme content between pigmented and non-pigmented strains were assessed using the Mann–Whitney U test. To test whether the isolation origin influenced functional genomic repertoires, the relationship between isolation source and (i) total CAZyme content and (ii) total number of BGCs was evaluated using the Kruskal–Wallis test. Statistical comparisons of the genomic distribution of pangenome components between primary and secondary chromosomes were performed using paired analyses. For each genome, the normalized abundance of core, accessory, and singleton gene families was calculated as the proportion of gene families relative to replicon size. Normality of paired differences between chromosomes was assessed using the Shapiro–Wilk test. Differences in the normalized abundance of core and accessory gene families between primary and secondary chromosomes were evaluated using the Wilcoxon signed-rank test, whereas singleton gene families were compared using a paired Student’s t-test. Statistical significance was considered at *p* < 0.05 for all analyses.

## Results

### Pangenome Structure and Lineage Diversification of *Pseudoalteromonas*

The genomes of 53 *Pseudoalteromonas* species averaged 4.85 Mb, ranging from 3.27 Mb (*P. qingdaonensis*) to 6.30 Mb (*P. obscura*). The GC content varied from 34.5% to 49.5%, with *P. denitrificans* exhibiting the lowest value within the genus (34.5%), consistent with its psychrophilic profile and adaptation to the cold waters of the Norwegian fjord (Supplementary Table S1).

The sources of isolation were diverse: 23 strains were recovered from marine organisms (including algae, mussels, sea cucumbers, shrimp, tunicates, sponges, corals, oysters, and fish), 21 from free-living seawater, and 9 from sediments. The lineages analyzed were collected from a wide range of marine environments, most were recovered from from the Pacific Ocean (28 lineages), followed by the Atlantic Ocean (15 lineages), the Antarctic Ocean (4 lineages), the Arctic Ocean (3 lineages), the Indian Ocean (2 lineages) and one lineage from Yundang Lake, a saltwater lake located in the center of Xiamen, China.

**Fig. 1:**
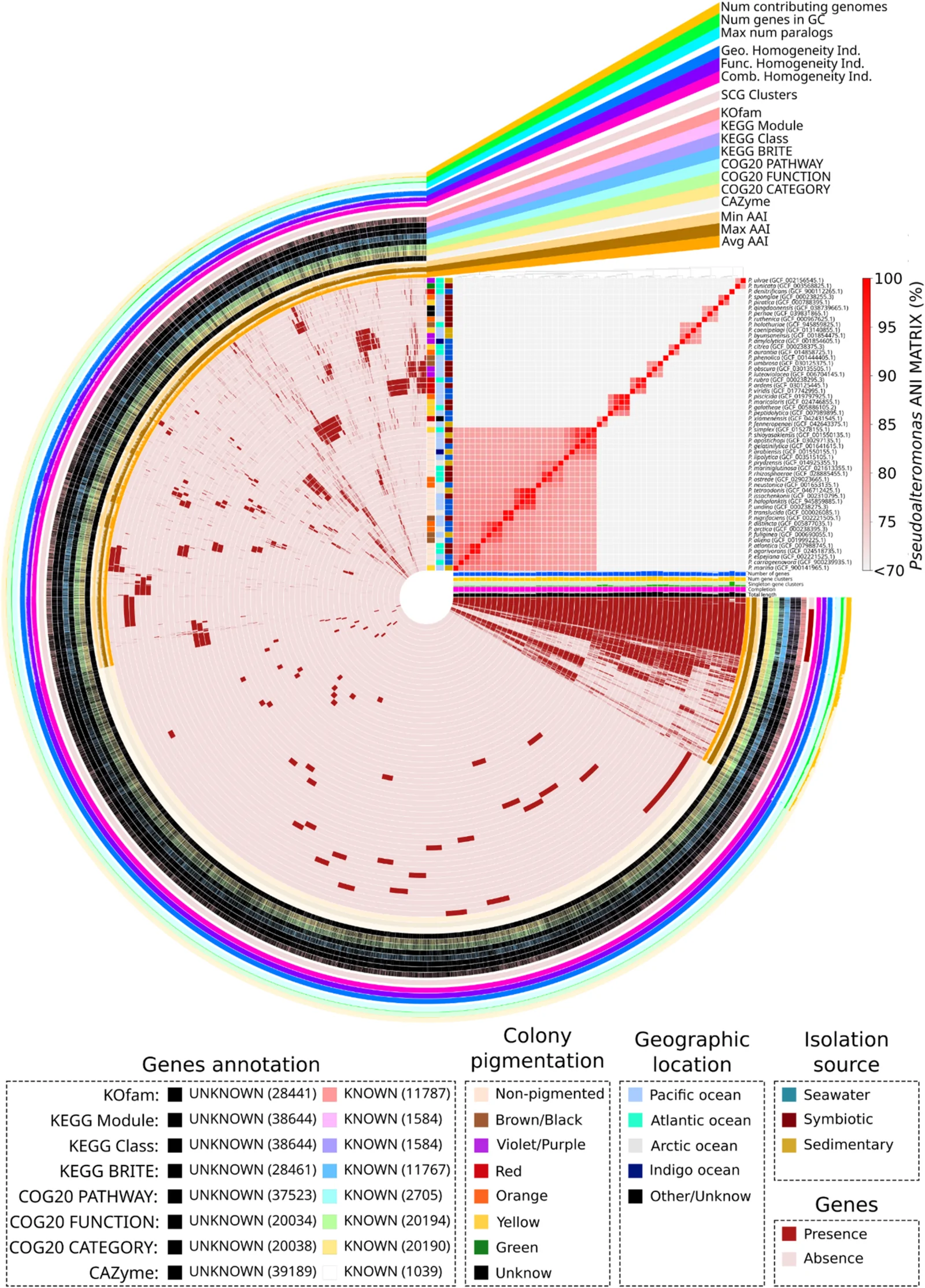
Pangenome analysis of 53 *Pseudoalteromonas* RefSeq genomes. The radial plot illustrates the distribution (presence/absence) of gene clusters among representative lineages of the genus. Each inner circular ring corresponds to a specific genome. The outer layers denote gene cluster metrics and annotations. In the upper right section, a heatmap displays the Average Nucleotide Identity (ANI) values across the genomes. Below it, bar charts summarize individual genomic metrics, including the total number of genes, gene clusters, singleton gene clusters, genome completion, and total genome length

In the context of the *Pseudoalteromonas* pangenome, pigmentation has traditionally been associated with the genus’s functional activities, including the production of bioactive metabolites and adaptation to diverse marine niches. In this analysis, 18 non-pigmented lineages and 33 pigmented lineages (yellow, orange, red, violet, green, or brown/grey) were identified, while pigmentation could not be determined for two additional lineages. A pattern of greater genomic proximity was observed among the non-pigmented lineages, while the pigmented lineages exhibited substantial divergence, both relative to the non-pigmented group and among themselves. Notably, some pigmented lineages clustered with non-pigmented ones (09 of 26), and, conversely, specific non-pigmented lineages grouped within pigmented clusters (01 of 27). This indicates that although coloration correlates with specific functional characteristics, it is not the sole determinant of phylogenetic proximity or lineage differentiation (Fig. 1). Although previous studies have linked the greater genomic variability of pigmented *Pseudoalteromonas* to diversification of secondary metabolism (Bosi et al. 2017), our findings indicate that pigmentation alone does not explain the observed genomic variability. This suggests that sequence divergence and accessory genome dynamics, rather than pigment production per se, underlie the genomic separation within the genus. Thus, multiple evolutionary forces, in addition to the biosynthetic capacity of secondary metabolites, contribute to the diversification of *Pseudoalteromonas*.

**Fig. 2:**
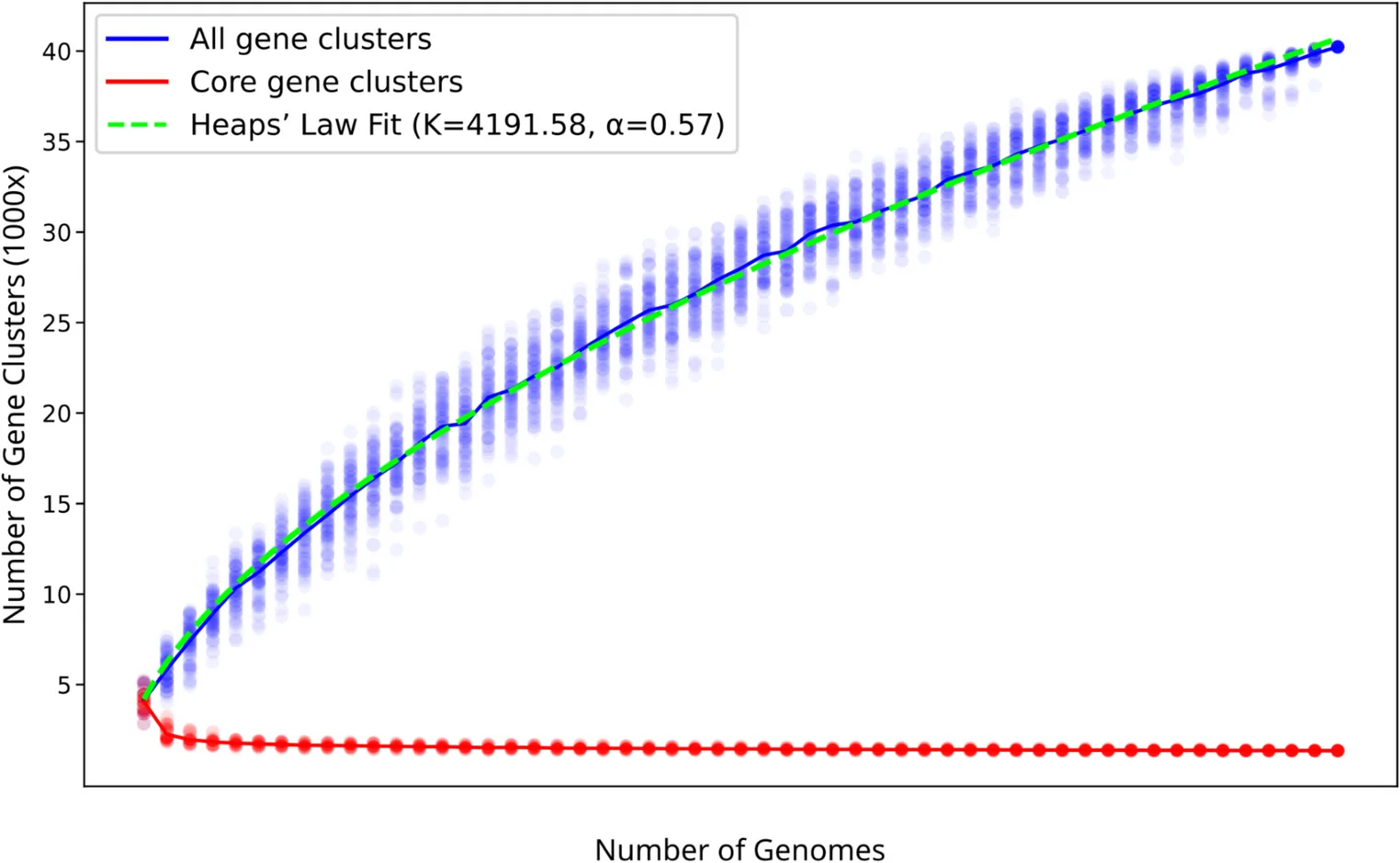
Heaps’ law model of the *Pseudoalteromonas* spp. pangenome. The model was calculated from 53 genomes. The gene accumulation curves for the pangenome and the core genome are shown in blue and red, respectively. The calculated α value indicates that the *Pseudoalteromonas* pangenome is open

**Fig. 3:**
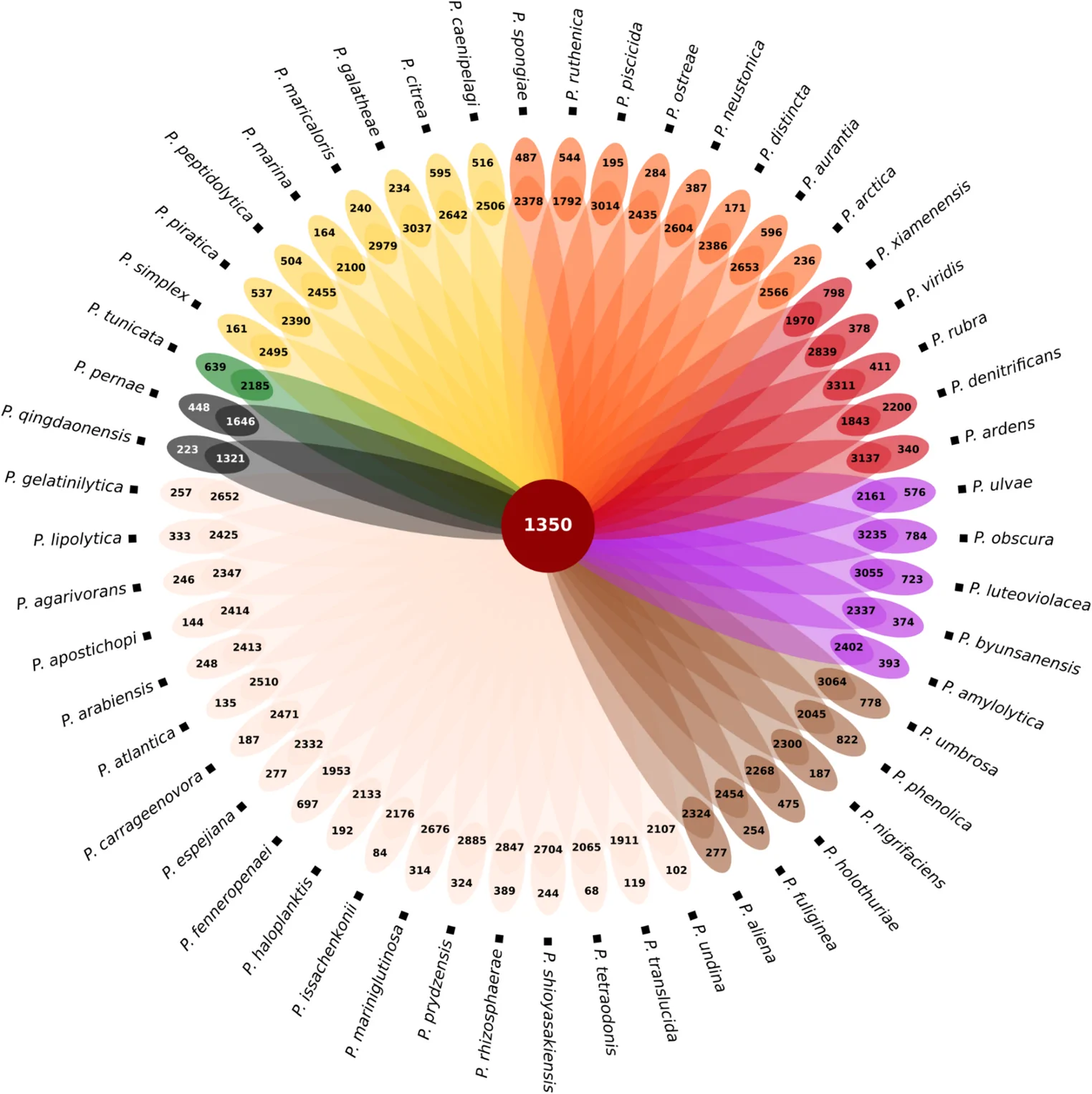
Distribution of genes in the genomes of *Pseudoalteromonas* spp. by flower plots showing the core gene number (in the center), accessory gene number (in the petals), and strain-specific gene number (at the tip of the petals) in 53 *Pseudoalteromonas* strains. The sum of the numbers shown beneath each strain represents the total number of gene families identified for that species. The colors of the petals represent the lineage's colony color. Petals in black represent lineages for which color descriptions are unavailable

Based on Fig. 2, Heap’s Law revealed a value of α = 0.57, indicating that the *Pseudoalteromonas* pangenome is open. The analysis revealed 40,228 gene clusters: 1,350 core clusters (3.36%), 18,115 accessory clusters (45.03%), and 20,763 strain-specific clusters (51.61%), representing the strain-specific gene content (Fig. 3). The individual genomes ranged from 2,894 to 5,393 genes, reflecting considerable variability in genome size and gene content within the genus. This contrast between the small conserved fraction and the large number of accessory genes reinforces the open nature of the pangenome and highlights the pronounced genomic plasticity of the genus.

Analysis of gene count distributions in the singleton and accessory categories was performed using the Shapiro-Wilk test. Singleton genes displayed a non-normal distribution (W = 0.70449, *p* < 0.001), whereas accessory gene counts did not significantly deviate from normality (W = 0.97702, *p* = 0.396), indicating an approximately normal distribution for the latter. The distribution of gene counts is further illustrated in Fig. S1, which presents violin plots with internal boxplots for each pangenome fraction. The accessory compartment exhibits consistently high values, with the highest density concentrated between ~ 2,000 and 2,700 genes, reflecting a stable distribution across genomes. In contrast, the singleton compartment shows substantially lower values, primarily below ~ 500 genes, but includes a notable outlier of 2,200 genes, representing a genome with an unusually high number of unique genes. Even after exclusion of the outlier genome containing 2,200 genes from the singleton category, the Shapiro-Wilk test indicated that the distribution remained non-normal (W = 0.933, *p* = 0.006). These patterns are consistent with continuous and widespread gene turnover across lineages, with occasional lineage-specific enrichment of unique genes.

**Fig. 4:**
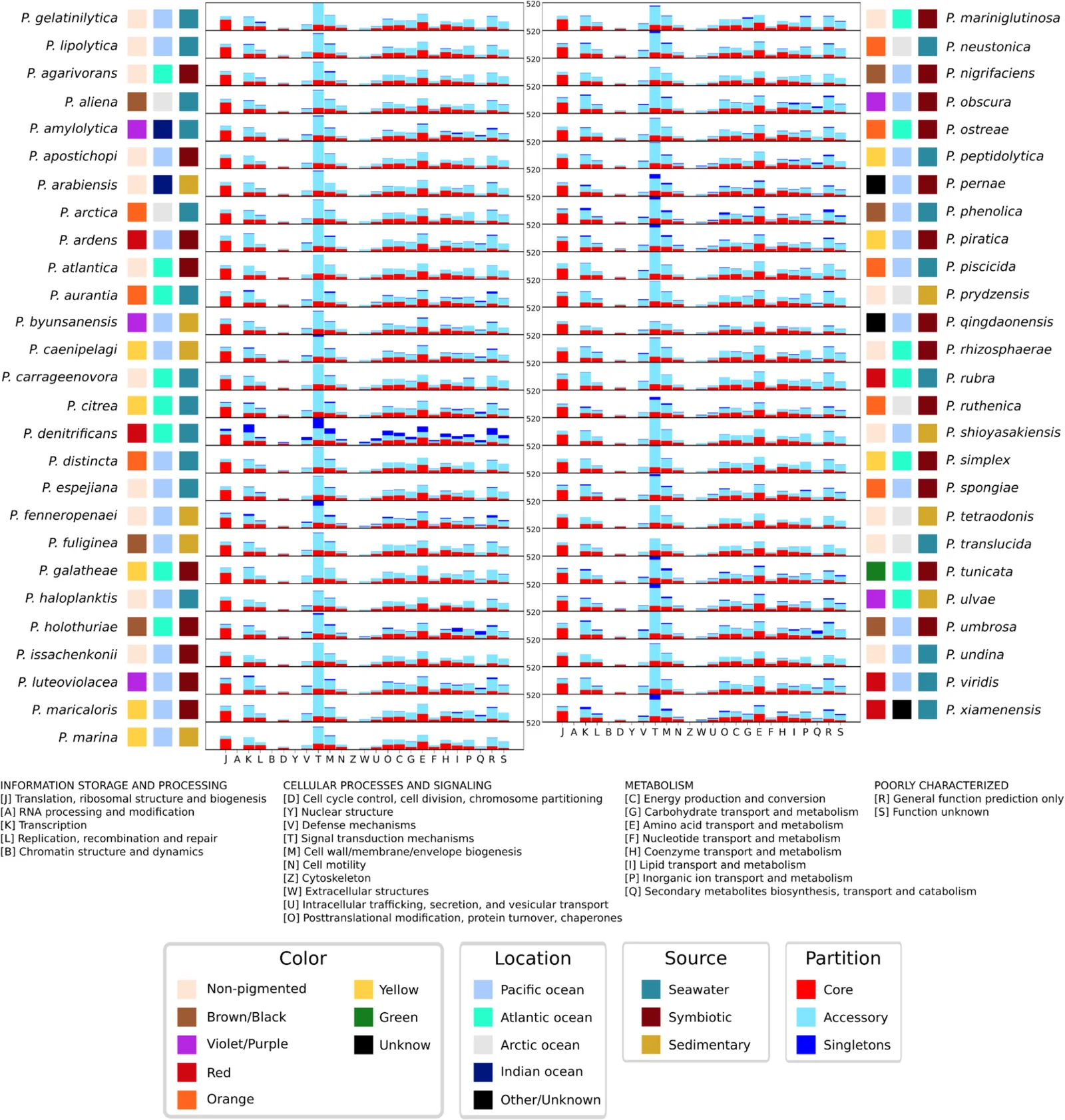
Functional classification of the *Pseudoalteromonas* pangenome partitions for each genome. Stacked bar charts illustrate the quantitative distribution of genes across COG functional categories. The bars color denote the pangenome partition to which the genes belong. Adjacent to each species name, three colored squares summarize strain metadata, indicating colony pigmentation, geographic location (ocean of origin), and environmental isolation source, respectively, as detailed in the bottom legends

Core genes were predominantly associated with essential cellular functions, including translation, ribosomal structure and biogenesis (15%), amino acid transport and metabolism (10%), coenzyme transport and metabolism (7%), energy production and conversion (6%), and several other housekeeping processes such as replication, recombination and repair, cell envelope biogenesis, transcription, and signal transduction (Fig. 4). In contrast, categories related to defense mechanisms (2%) and secondary metabolite biosynthesis, transport and catabolism (1%) were comparatively underrepresented within the core genome. Notably, *P. denitrificans* stands out due to a higher number of singleton genes across nearly all COG categories, further supporting its phylogenetic distinctiveness and suggesting the presence of lineage-specific adaptive traits (Fig. 4).

### Core Genes

The pangenome of the genus *Pseudoalteromonas* reveals a relatively small core (1350 orthologous coding sequences), in which essential functions are highly conserved, in contrast to the accessory fraction, which concentrates most of the genetic variability. Although the functional composition is relatively similar between species, genomic differentiation is primarily driven by variation within the accessory genome.

**Fig. 5:**
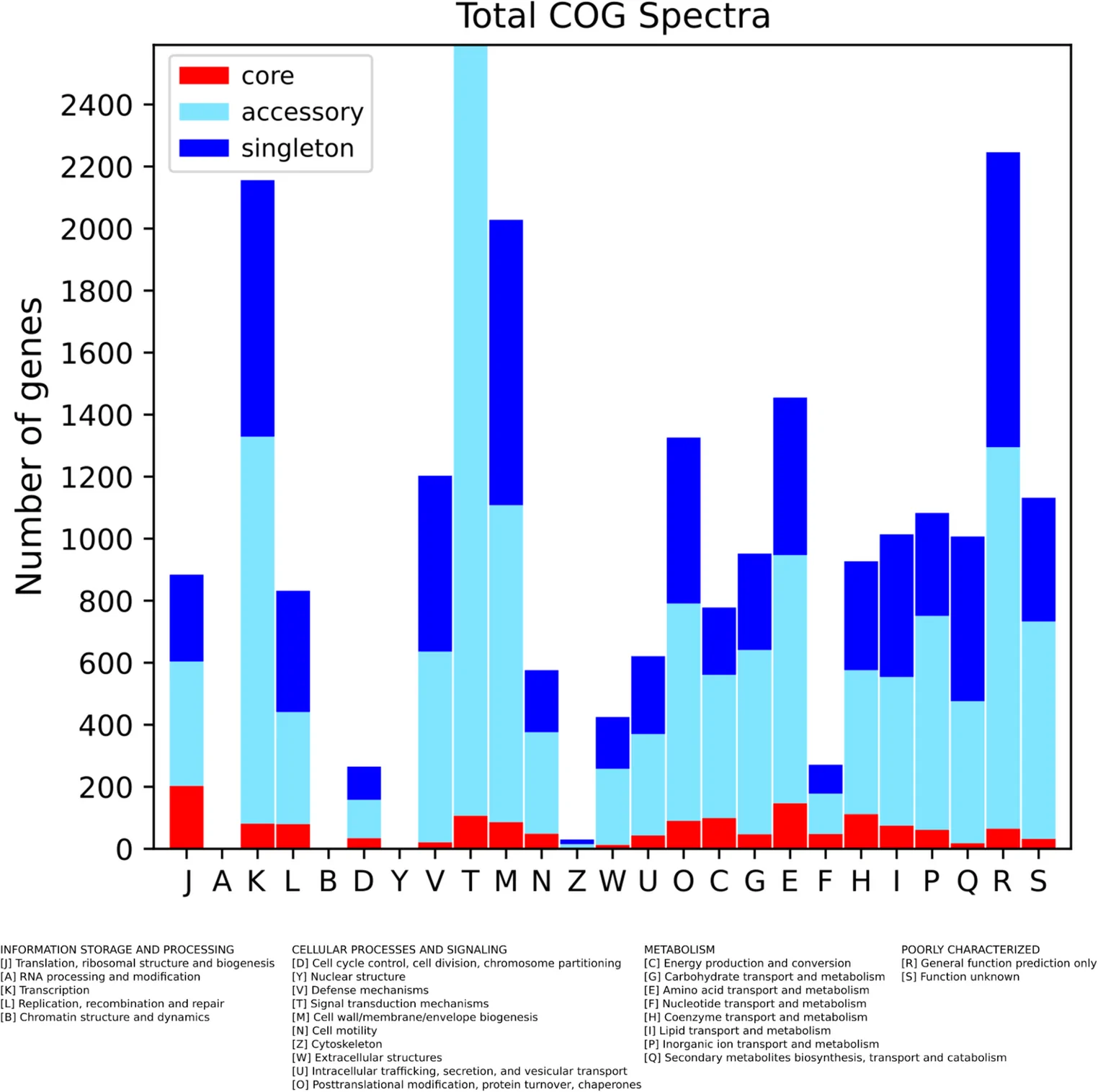
Distribution of genes by COG functional category in the *Pseudoalteromonas* pangenome. The stacked bar chart displays the absolute number of genes assigned to each COG functional category across the entire pangenome. Bar colors illustrate the proportion of the different pangenome partitions for each category

Results from the core genes of *Pseudoalteromonas* indicate that approximately 40% of gene families are associated with metabolism, and a notable feature is that genes responsible for translation, ribosomal structure, and biogenesis account for approximately 15% of the core genes (Fig. 5). The conservation of these functions across all genomes likely reflects the need to maintain efficient protein synthesis across the diverse ecological niches occupied by *Pseudoalteromonas*, ranging from tropical to polar marine environments. This pattern supports the existence of a conserved functional backbone that remains stable despite the high genomic plasticity and ecological diversification observed within the genus. This variety of gene families specialized for translation enables fast and efficient gene expression, consistent with their roles as opportunistic surface colonizers and in symbiotic interactions. Among these genes, we can highlight *groEL*,* groES*,* dnaK*,* dnaJ*,* and tig (Trigger factor)*, which act as chaperones that support protein folding. These chaperone systems contribute to protein homeostasis and efficient protein synthesis across the broad range of temperature or marine environments occupied by *Pseudoalteromonas*, including tropical, temperate, Arctic, and Antarctic habitats. There are still 131 gene families of poorly characterized (unknown function, general function prediction only or no function class found) in the core genome, comprising 10% of all core genes (Fig. 5). Although this category represents a relatively small fraction of the other functional classes, this finding is consistent with growing research on the genus, which has progressively elucidated its survival and adaptive mechanisms.

### Accessory Genes

In contrast to the conserved core genome, the accessory genome comprises genes that are variably distributed among strains and often reflects ecological adaptation and functional diversification (Fig. 5). In marine bacteria, accessory genes frequently encode traits involved in environmental interactions, including the production of pigments and specialized metabolites that enhance survival under diverse environmental conditions.

*P. qingdaonensis*, isolated from the intestine of the crab *Ilyoplax deschampsi* in Qingdao, China, exhibits a relatively small genome and the lowest number of accessory genes within the genus (1,321 genes). Notably, this species shows a marked reduction in gene families involved in transcription and signal transduction, particularly within the accessory non-singleton fraction. This genomic profile likely reflects ecological specialization to a restricted niche, where essential metabolic functions are sufficient for survival and the acquisition of additional genes is unnecessary (Moran 2002). Genome compactness and reduced accessory gene content illustrate how local selective pressures and environmental stability can shape genomic composition in species adapted to specific habitats.

In contrast, *P. rubra* possesses the largest number of accessory genes (3,311 genes), a pattern also observed in other pigmented species such as *P. obscura* (3,235 genes) and *P. ardens* (3,137 genes). Notably, all three species exhibit pigmentation and harbor more than 3,100 accessory genes, more than twice the number observed in *P. qingdaonensis.*

In the most conserved *Pseudoalteromonas* cluster, comprising 26 lineages phylogenetically related to non-pigmented strains (Fig. 1), approximately 29% of COGs are classified as poorly characterized, highlighting a substantial potential for functional discovery. Among the annotated COG categories, 24% are related to metabolism, 32% to cellular processes and signaling, and 6% to information storage and processing. Notably, enriched functions include those associated with primary metabolism, replication, recombination, and DNA repair, as well as post-translational modification, protein turnover, and chaperone activity. This functional profile reflects a robust network dedicated to stress response and genomic maintenance, supporting cellular integrity and stability. The enrichment of these functions may partially offset the lower representation of genes associated with motility and secondary metabolite production, potentially contributing to ecological persistence under fluctuating marine environmental conditions.

### Singleton Genes

Among the 20,763 singleton genes identified across the *Pseudoalteromonas* genomes, only 7,717 (37.2%) could be assigned to functional categories, whereas 13,046 genes (62.8%) remained unclassified. This high proportion of unannotated singleton genes reveals an extensive reservoir of poorly characterized genetic diversity within the genus and suggests that a substantial fraction of lineage-specific functions remains unexplored. Such a pattern is commonly observed in accessory genomes, where recently acquired or niche-specific genes are often underrepresented in current functional databases.

Within the fraction of singleton genes that could be annotated, only a subset was assigned to known functional categories. The most abundant categories were cell wall/membrane/envelope biogenesis (9.6%), transduction mechanisms (9.0%), general function prediction only (8.5%), transcription (6.3%), and defense mechanisms (6.1%). Additional contributions were observed for posttranslational modification, protein turnover and chaperones (4.3%), replication, recombination and repair (4.0%), inorganic ion transport and metabolism (3.6%), amino acid transport and metabolism (3.2%), and carbohydrate transport and metabolism (3.0%). This distribution suggests that *Pseudoalteromonas* strain-specific genes are predominantly associated with environmental interaction, perception and response to external stimuli, remodeling of the cell surface, defense strategies, and nutrient acquisition and utilization.

Notably, a considerable fraction of the annotated singletons was assigned to general function prediction only (8.5%) or function unknown (4.7%). In addition, the occurrence of defense mechanisms (6.1%), replication and recombination functions (4.0%), and mobilome-associated genes (2.2%) suggests that genome plasticity and horizontal gene transfer contribute to the acquisition of lineage-specific traits. Overall, the relatively small proportion of genes involved in secondary metabolite biosynthesis, transport and catabolism (2.7%) indicates that ecological diversification in *Pseudoalteromonas* is likely driven not only by specialized metabolism but also by regulatory, structural, and defense-related adaptations.

### Genomic Distribution of Pangenome Components in Multipartite *Pseudoalteromonas* Genomes

Among the 53 genomes included in the pangenome analysis, only 17 were classified as complete genomes according to the NCBI database and were therefore suitable for investigating gene family distribution across multipartite genomes (Fig. S2). Of these, 16 exhibited a multipartite architecture consisting of two or three replicons, whereas only *P. aliena* possessed a single chromosome. Analysis of core gene family distribution revealed a strong concentration within the primary chromosome. Of these, 16 exhibited a multipartite architecture, with 12 genomes containing two replicons and only four containing three replicons, whereas *P. aliena* possessed a single chromosome. These findings indicate that the conserved genomic repertoire of *Pseudoalteromonas* is predominantly maintained within the primary chromosome.

To account for differences in replicon size, core, accessory and singleton gene families abundance was normalized by replicon length. The primary chromosome exhibited an average core gene family enrichment approximately 3.3-fold higher than that observed in secondary replicons, demonstrating that the predominance of core genes is not merely a consequence of chromosome size. This pattern suggests a functional partitioning of the multipartite genome, whereby the primary chromosome preferentially retains conserved and essential functions, while secondary replicons contribute proportionally less to the core genome. The near absence of core genes in tertiary replicons further suggests that these elements behave more similarly to accessory genetic compartments, potentially providing genomic space for adaptive and lineage-specific traits.

In contrast to the distribution observed for core gene families, accessory gene families were enriched in secondary replicons. After excluding a small outlier replicon from *P. rhizosphaerae*, the normalized abundance of accessory genes averaged 0.059 ± 0.011% in primary chromosomes and 0.168 ± 0.044% in secondary replicons. Thus, accessory genes were approximately 2.8-fold more enriched in secondary replicons than in primary chromosomes. Moreover, the higher dispersion observed among accessory gene densities compared with core genes suggests greater variability in the accessory genome across species.

Singleton gene families exhibited a more heterogeneous distribution across replicons compared to core and accessory genes. The normalized abundance of singletons ranged from approximately 0.002% to 0.015% in primary chromosomes and from 0.001% to 0.023% in secondary replicons, indicating substantial variability among genomes. On average, singleton genes were only slightly enriched in secondary replicons (~ 1.4-fold), contrasting with the stronger compartmentalization observed for core and accessory genes.

Notably, singleton gene abundance displayed considerably higher dispersion than that observed for core and accessory gene families, suggesting a more stochastic distribution influenced by recent gene acquisition events. In a subset of genomes containing small third replicons, singleton genes were enriched, reaching values up to 0.127%, indicating that these replicons may serve as hotspots for lineage-specific innovation.

To further evaluate the genomic partitioning of multipartite genomes, the distribution of core, accessory, and singleton gene families was compared between primary and secondary chromosomes across the 16 complete *Pseudoalteromonas* genomes. Paired Wilcoxon signed-rank tests revealed significant differences for all three pangenome components. Core gene families were significantly enriched in primary chromosomes (*p* = 0.00048), supporting the role of the main chromosome as the principal repository of conserved and essential functions. In contrast, accessory gene families were significantly enriched in secondary chromosomes (*p* = 0.00071), consistent with the hypothesis that secondary replicons function as reservoirs of adaptive genetic content and contribute disproportionately to pangenome expansion. Singleton gene families exhibited a modest but significant enrichment in secondary chromosomes (paired t-test, *p* = 0.022; Cohen’s d = 0.64), supporting the view that secondary replicons contribute disproportionately to the accumulation of lineage-specific genes. Together, these results support a model in which multipartite genomes of *Pseudoalteromonas* are organized along a continuum of genetic stability and innovation, with core genes concentrated in the primary chromosome, accessory genes enriched in secondary replicons, and singleton genes exhibiting the highest variability between chromosomes, but improve in second chromosome and association with smaller replicons.

### CAZymes repertoires across *Pseudoalteromonas* Strains

**Fig. 6:**
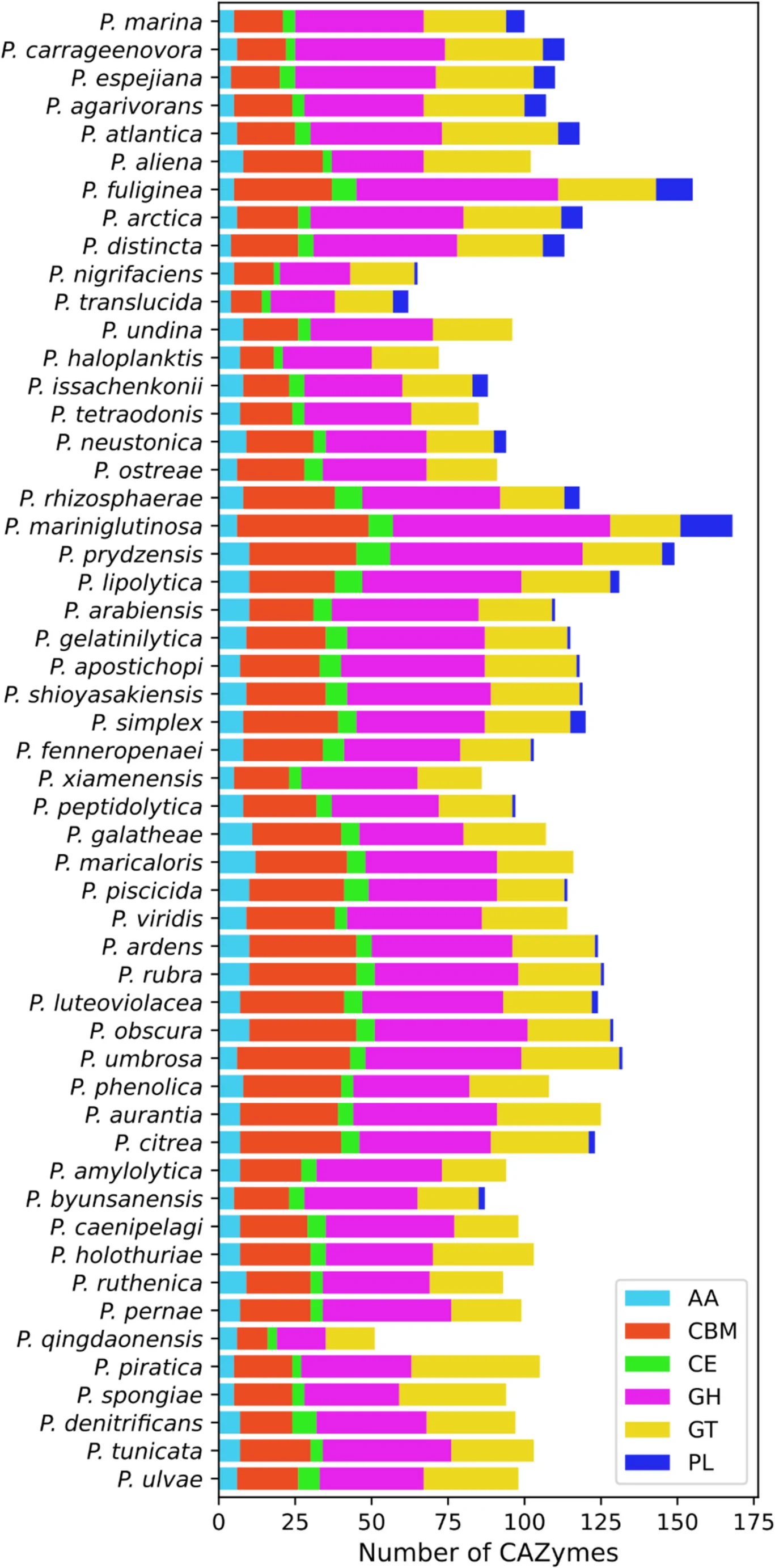
Carbohydrate-active enzyme repertoires across *Pseudoalteromonas* Strains. The distribution of carbohydrate-active enzymes (CAZymes) families by strain of *Pseudoalteromonas* were classified according to the CAZy database into the following classes: AA (Auxiliary Activities), CBM (Carbohydrate-Binding Modules), CE (Carbohydrate Esterases), GH (Glycoside Hydrolases), GT (GlycosylTransferases), and PL (Polysaccharide Lyases)

Among CAZymes, the highest gene counts were observed in are *P. mariniglutinosa* (168 genes), *P. fuliginea* (155), and *P. prydzensis* (149), all belonging to the non-pigmented or brown lineage (Fig. 6). These analyses are framed within an exploratory approach aimed at assessing potential associations between metabolic capacity and ecological and phenotypic traits, rather than inferring direct causal relationships. However, no statistically significant difference in overall CAZyme abundance was detected between pigmented and non-pigmented strains (Wilcoxon rank-sum test, W = 313.5, *p* = 0.75). These species therefore represent individual cases occupying the upper extreme of the CAZyme distribution rather than a general pattern associated with pigmentation.

**Fig. 7:**
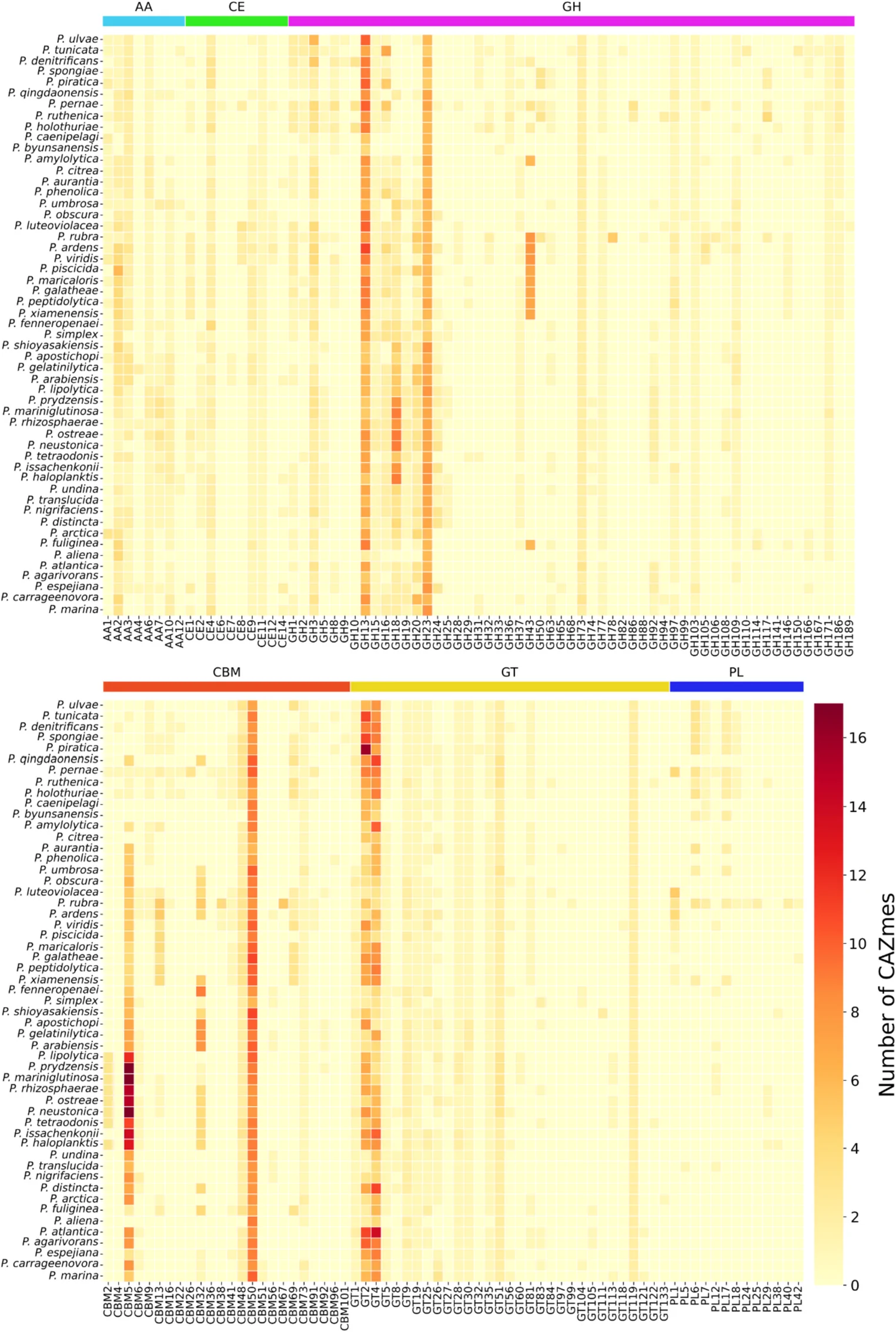
Distribution and abundance of CAZymes in the *Pseudoalteromonas* pangenome. The heatmap illustrates the number of genes assigned to CAZyme families in each strain of *Pseudoalteromonas.* These families are grouped into six major classes, indicated by the colored bars at the top of each panel: AA (Auxiliary Activities), CBM (Carbohydrate-Binding Modules), CE (Carbohydrate Esterases), GH (Glycoside Hydrolases), GT (GlycosylTransferases), and PL (Polysaccharide Lyases)

The distribution of CAZy families varies across lineages, ranging from 4 to 12 genes for auxiliary activities (AA), redox enzymes that act in conjunction with CAZymes; 10–43 for carbohydrate-binding modules (CBM), adhesion to carbohydrates; 2–11 for carbohydrate esterases (CE), hydrolysis of carbohydrate esters; 16–71 for glycoside hydrolases (GH), hydrolysis and/or rearrangement of glycosidic bonds and 16–42 for glycosyltransferases (GT), formation of glycosidic bonds. Polysaccharide lyases (PL), non-hydrolytic cleavage of glycosidic bonds, constitute the only CAZy family not universally present among all genomes, with copies numbers varying from 0 to 17 genes. The GT group, which represents the most enriched among all CAZy families, includes genes such as GT2 (1–16 copies), GT4 (2–14), GT9 (1–2), GT28 (1–2), GT30 (1–2), and GT251 (2–3). Notably, GT119 stands out, as all lineages harbor two copies of this gene. Within the GH family, GH13 (2–11 copies), GH23 (4–8 copies) and GH73 (2–3 copies) are the most abundant and are consistently present across all analyzed genomes (Fig. 7).

Based on the CAZy database definitions, the predicted enzyme families GH23 and GH73 are associated with lysozyme-like folds. GH23 enzymes act as peptidoglycan lytic transglycosylases, cleaving peptidoglycan without the involvement of water, whereas the β-1,4-N-acetylmuramoylhydrolase activity of GH73 has not yet been confirmed. The GH13 family is the most prominent glycoside hydrolase family described to date, comprising enzymes that cleave glycosidic bonds and act primarily in starch hydrolysis, modification, or transglycosylation (Graebin et al. 2016).

### Distribution and Diversity of BGCs of *Pseudoalteromonas* Strains

**Fig. 8:**
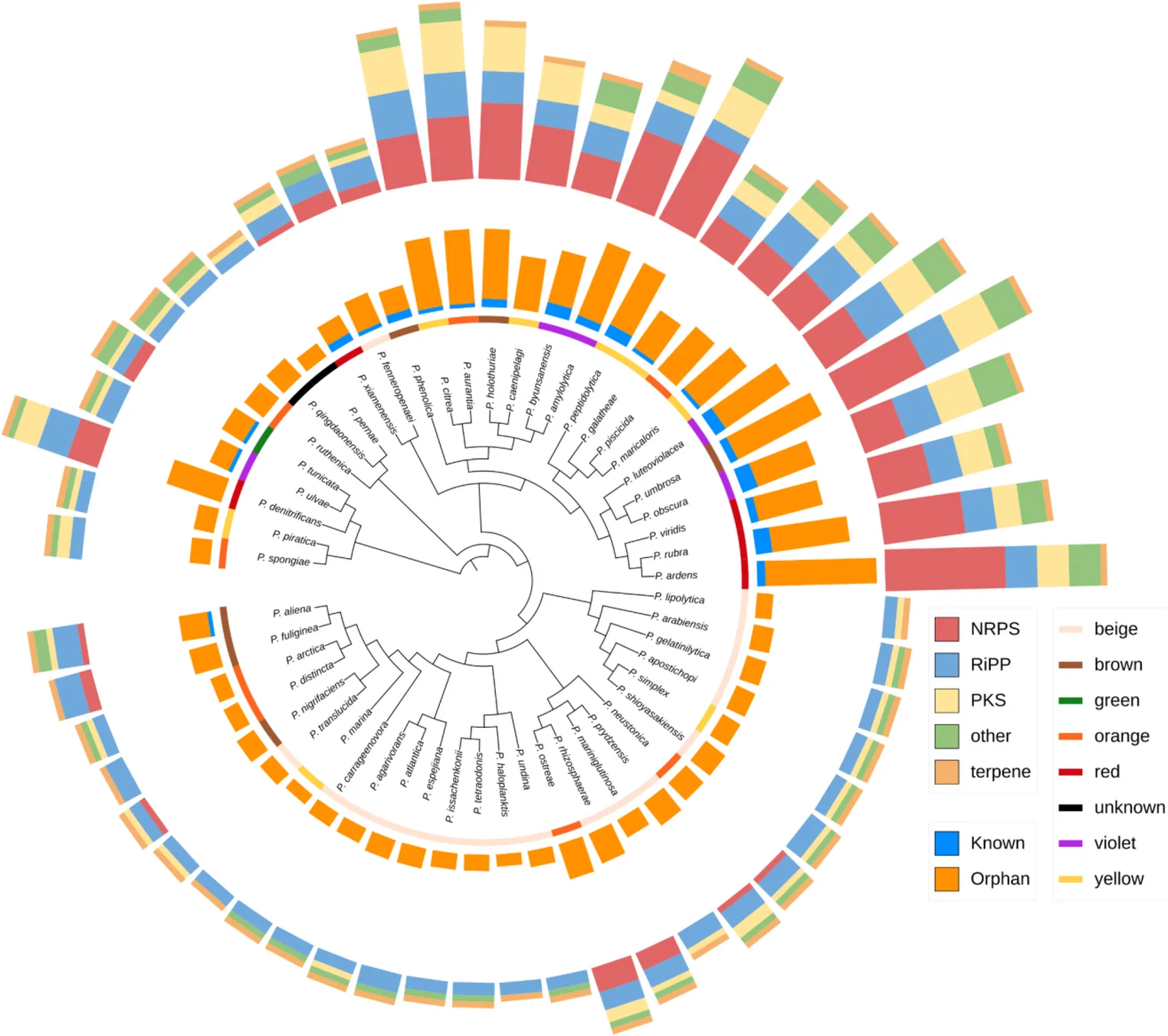
Phylogenomic and biosynthetic potential analysis of the genus *Pseudoalteromonas*. At the center, a circular phylogenomic tree details evolutionary relationships among *Pseudoalteromonas* species. The inner colored ring indicates the colony pigmentation observed for each species. Concentric rings of stacked bar charts summarize Biosynthetic Gene Cluster (BGC) data for each genome. The inner bar chart displays the distribution of known and orphan BGCs per genome, while the outer chart displays the biosynthetic pathway class composition of these BGCs. NRPS: Non-Ribosomal Peptide Synthetase; RiPP: Ribosomally synthesized and Post-translationally modified Peptides; PKS: Polyketide Synthase

Figure 8 shows the phylogenomic distribution and its association with pigmentation and BGC content. Phylogenetically diverse species are more strongly associated with pigmentation and harbor a higher proportion of BGCs, mostly orphan clusters. The number of BGCs identified in *Pseudoalteromonas* genomes using antiSMASH ranged from 3 to 29 per genome, reflecting considerable variation in secondary metabolite potential across the genus. While some pigmented lineages exhibited a BGC count comparable to non-pigmented lineages, all genomes containing more than 10 BGCs corresponded exclusively to pigmented strains. This observation indicates that pigmentation may be associated with an increased biosynthetic potential. These analyses are framed within an exploratory comparative framework aimed at assessing potential associations between biosynthetic capacity, phylogenetic relatedness, and ecological/phenotypic traits, rather than inferring direct causal relationships.

**Fig. 9:**
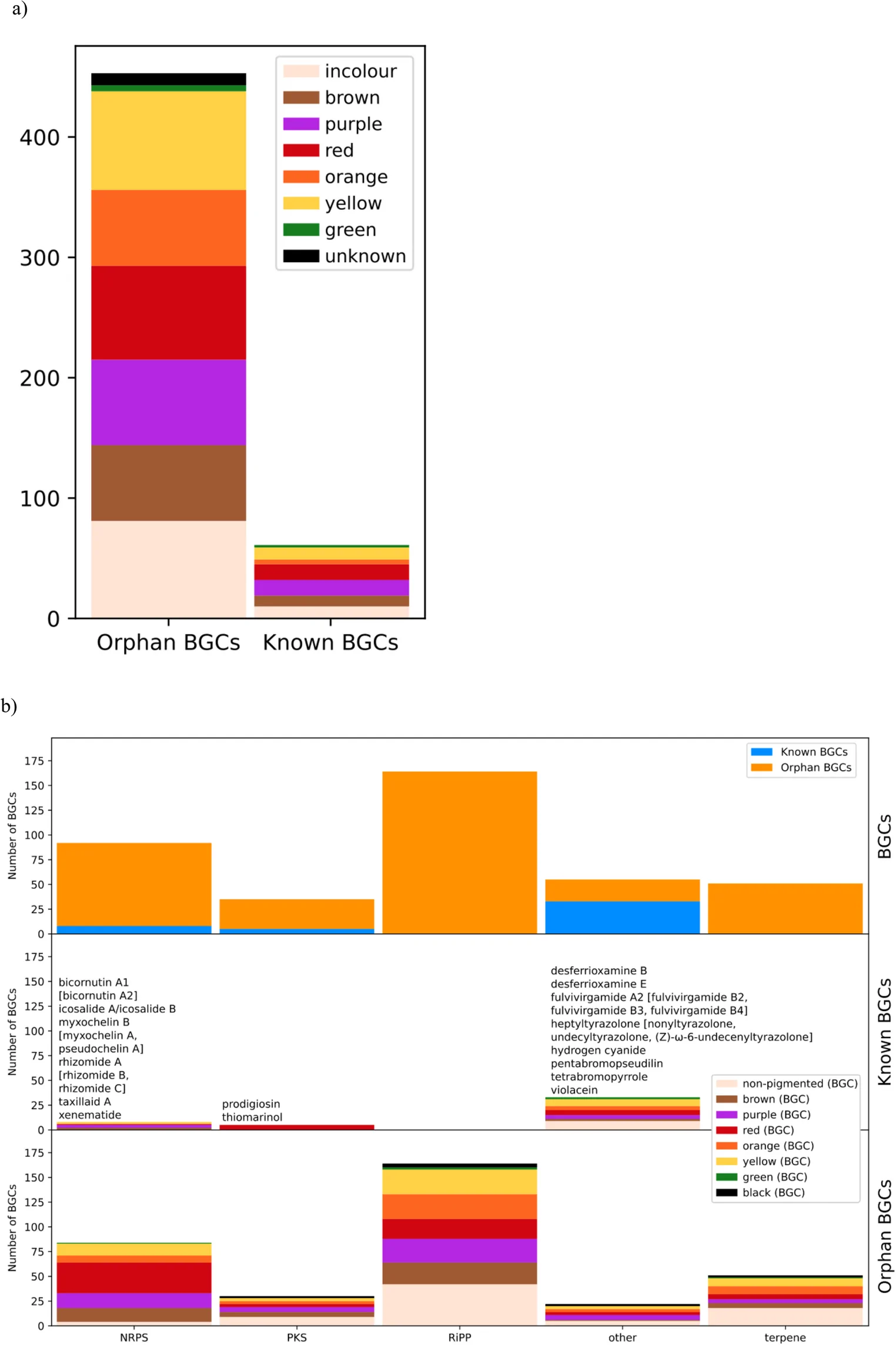
Biosynthetic Gene Clusters (BGCs) of the *Pseudoalteromonas* pangenome. (**a**) The left panel displays the absolute number of orphans and known BGCs, based on the antiSMASH results. The right panel illustrates the distribution of these orphan and known BGCs across the different colony pigmentation phenotypes of the source species. (**b**) Distribution of Biosynthetic Gene Clusters (BGCs) across major biosynthetic classes in the *Pseudoalteromonas* pangenome. The top panel illustrates the total number of predicted BGCs per class, partitioned into orphan and known clusters. The middle and bottom panels detail the distribution of these known and orphan BGCs, respectively, highlighting the colony pigmentation of the source species. In the middle panel, the known secondary metabolites predicted by antiSMASH are listed above their corresponding biosynthetic class bars. NRPS: Non-Ribosomal Peptide Synthetase; PKS: Polyketide Synthase; RiPP: Ribosomally synthesized and Post-translationally modified Peptides

Interestingly, the majority of BGCs identified in *Pseudoalteromonas* genomes correspond to orphan clusters, with only a minor fraction being functionally characterized or previously described, except for the ‘other’ class, which accounts for the largest proportion of known BGCs (Fig. 9). This observation underscores the largely unexplored potential for secondary metabolites within the genus and highlights opportunities to discover novel bioactive compounds. Interestingly, among BGC classes, post-translationally synthesized and modified ribosomal peptides (RiPPs) were the most prevalent in the genomes studied, although none of these clusters have been functionally characterized to date.

BiG-SCAPE analysis grouped the identified BGCs into 56 gene cluster families (GCFs), each comprising at least two BGCs. Of these, 14 GCFs were associated with known or predicted products, including clusters related to prodigiosin (*n* = 5), violacein (*n* = 7, distributed across two clusters), tambjamine (*n* = 4), arylpolyenes (*n* = 25, across three clusters), hydrogen cyanide (*n* = 14, across two clusters), and polyhalogenated pyrroles, predicted as pentabromopseudilin or tetrabromopyrrole (*n* = 7). Additional assignments included a siderophore-related predicted to encode putrebactin, avaroferrin, deferrioxamine, or fulvivirgamide (*n* = 28), as well as GCFs associated with rhizomide (*n* = 3), myxochelin/pseudochelin (*n* = 3), and hserlactone-type compounds, including tyrazolone derivatives (*n* = 2).

The remaining 42 BGCs lacked confident annotation and were therefore classified as orphan clusters. These were predominantly represented by RiPPs (*n* = 9 families; 155 BGCs), NRPS (*n* = 18; 44 BGCs), PKS (*n* = 2; 5 BGCs), terpenes (*n* = 1; 50 BGCs), and other classes (*n* = 3; 10 BGCs). In addition, nine orphan families exhibited hybrid architectures, including NRPS-PKS (*n* = 7; 37 BGCs), NRPS-RiPP (*n* = 1; 4 BGCs), and NRPS-PKS-other (*n* = 1; 8 BGCs). Notably, 114 BGCs identified by antiSMASH did not cluster in the BiG-SCAPE network, suggesting a high degree of uniqueness within the dataset (Fig. S3).

### Linking Pigmentation and Isolation Origin to Functional Genomic Repertoires

Despite lineage-specific expansions in functional repertoires, statistical analyses did not reveal a significant association between pigmentation and either CAZyme abundance or overall functional composition, indicating that diversification of carbohydrate-active enzyme repertoires within the genus is not primarily constrained by pigmentation.

Nonetheless, pigmented genomes harbored a significantly higher number of BGCs compared to non-pigmented lineages (Mann–Whitney test, *p* < 0.001; Fig. S5). This result suggests that pigmentation is associated with increased biosynthetic potential; however, it does not constitute a definitive predictor of the secondary metabolite repertoire across *Pseudoalteromonas*.

In this context, a weak but statistically significant positive correlation was observed between the number of BGCs and the total CAZyme content (Spearman’s ρ = 0.31, *p* = 0.0238; Fig. S5). This pattern suggests that genomes with greater secondary metabolite biosynthetic potential may also harbor a slightly broader repertoire of carbohydrate-active enzymes.

At the species level, Fig. S4 illustrates the distribution of CAZyme content and BGCs across *Pseudoalteromonas*. No consistent pattern links high CAZyme abundance to elevated BGC counts, supporting the weak correlation observed at the genomic level. Differences among species indicate variability in functional repertoires, with distinct combinations of CAZyme families and proportions of known and orphan BGCs. These results reinforce that carbohydrate metabolism and secondary metabolite biosynthesis are not uniformly associated across the genus.

To assess whether environmental origin influences the functional genomic landscape of *Pseudoalteromonas*, we further tested the association between isolation source and (i) total CAZyme content and (ii) the number of BGCs predicted by antiSMASH using the non-parametric Kruskal–Wallis test. No significant differences were detected among isolation categories for either variable. CAZyme abundance did not differ significantly according to isolation origin (χ² = 0.188, df = 2, *p* = 0.910), and similarly, BGC counts showed no significant association with isolation source (χ² = 0.283, df = 2, *p* = 0.868) (Fig. S6).

Taken together, these results indicate that, at the genus level, functional potential related to carbohydrate metabolism and secondary metabolite biosynthesis is not primarily structured by the isolation environment. Instead, these traits appear to be more strongly shaped by phylogenetic relationships, lineage-specific expansions, and ecological interactions that are not fully captured by broad isolation-source categories.

To further evaluate the robustness of the observed associations, we reassigned brown-pigmented lineages to the non-pigmented strains category and repeated the statistical analyses. This reclassification did not substantially alter the overall results, as no significant association was detected between pigmentation and CAZyme abundance (Wilcoxon rank-sum test, W = 313.5, *p* = 0.75), while the relationship between pigmentation and BGC content remained consistent (Wilcoxon rank-sum test, *p* < 0.001). Additionally, the previously observed weak relationship between CAZyme content and BGC number remained marginal, indicating a weak positive association that did not reach statistical significance (Spearman’s ρ = 0.27, *p* = 0.054). Instead, it may reflect the presence of conserved metabolic features, such as melanin-like compounds, which are often associated with core cellular functions rather than lineage-specific BGCs. Consequently, the inclusion or exclusion of brown-pigmented lineages does not significantly impact the broader functional patterns observed across the genus.

## Discussion

### Pigmentation as a Marker of Biosynthetic

The association observed between pigmentation and a higher number of BGCs suggests that pigment production in *Pseudoalteromonas* is embedded within a broader context of metabolic specialization. Bacterial pigments are often linked to ecologically relevant functions, including chemical defense, microbial competition, and protection against environmental stressors, which are commonly mediated by secondary metabolites (Chatragadda and Dufossé 2021). Therefore, the enrichment of BGCs in pigmented genomes may reflect shared selective pressures associated with niche adaptation, particularly in competitive and chemically dynamic marine environments. In this context, our findings support an association between pigmentation and enhanced biosynthetic capacity, although this does not imply a direct role in genome-wide diversification. Rather, pigmentation appears to be associated with broader metabolic specialization and ecological adaptation. Thus, multiple evolutionary forces, in addition to the biosynthetic capacity of secondary metabolites, contribute to the diversification of *Pseudoalteromonas*. A similar phenomenon has been observed in *Synechococcus*, where differences in pigmentation genes do not always result in consistent separations across the genome (Grébert et al. 2022).

Building on this perspective, our results further reinforce this hypothesis. In addition to harboring an abundance and diversity of BGCs, pigmented strains genomes also exhibited lower average nucleotide identity (ANI) in comparison to non-pigmented strains, indicating higher genomic divergence (Fig. 1). This differentiation is also evident in the phylogenomic tree, where pigmented lineages tend to cluster in more distinct and phylogenetically dispersed branches (Fig. 8). Such patterns suggest that these strains have undergone distinct evolutionary histories, potentially reflecting adaptation to different ecological contexts. Altogether, the concordance between phylogenomic structuring, reduced genomic similarity, and BGC enrichment supports the idea that pigmentation is associated with broader metabolic specialization and ecological differentiation within the genus *Pseudoalteromonas*. Among BGC classes, post-translationally synthesized and modified ribosomal peptides (RiPPs) were the most prevalent in the genomes studied, although none of these clusters have been functionally characterized to date. This observation aligns with global analyses of marine bacterial genomes, in which RiPPs are the second most common class of BGCs (21%), after terpenoids (24%) (Moghaddam et al. 2021) (Fig. 9). In ocean microbiome cluster families, much of the newly identified diversity also comprises predicted RiPPs, particularly in surface waters and deeper photic zones, and in underexplored habitats such as polar regions and the deep ocean (Paoli et al. 2022). However, only a few marine bacterial RiPPs, from both cultured and uncultured bacteria, have been experimentally characterized in the last decade, indicating that many BGCs remain to be explored. Advances in genome mining, metabolic engineering, and synthetic biology are expected to enable the discovery and functional characterization of these RiPPs, expanding their potential for future applications (Sukmarini 2022).

The genomic basis of pigmentation in *Pseudoalteromonas* further supports the link between accessory genome content and metabolic potential. Specific pigments, such as prodigiosin and violacein, showed a clear association with lineage-specific BGCs, indicating that certain phenotypes can be directly traced to discrete genetic determinants. In contrast, yellow and orange phenotypes were not consistently associated with unique or conserved BGCs, suggesting that these colorations may result from combinatorial pathways, regulatory effects, or structural variation in metabolites rather than single biosynthetic loci. Brown pigmentation, however, appears to be independent of canonical BGCs and is likely associated with melanin production, whose genetic potential is broadly conserved across lineages and whose phenotypic expression depends on regulatory and environmental conditions. The presence of pigment-related clusters in both pigmented and non-pigmented strains further indicates that gene presence alone is not sufficient to predict phenotype. Together, these patterns highlight that pigmentation reflects different levels of genomic and regulatory complexity and reinforces the role of the accessory genome in shaping phenotypic variability relevant to ecological interactions and bioprospecting.

The red colony coloration observed in certain *Pseudoalteromonas* strains is attributed to the presence of prodigiosin, a biosynthetic gene cluster BGC found exclusively in red-pigmented lineages and present in all strains exhibiting this coloration. This finding establishes a direct link between a specific secondary metabolite and the observed pigment, underscoring the ecological and biotechnological significance of prodigiosin. Notably, prodigiosin has been reported to exhibit antimicrobial activity against marine pathogens, such as *Vibrio harveyi*, as demonstrated in *P. viridis* BBR56 (Handayani et al. 2024).

The purple pigmentation is associated with the presence of the violacein biosynthetic gene cluster (BGC). Violacein is an indole-derived compound synthesized from two L-tryptophan molecules (Balibar and Walsh 2006). In *Pseudoalteromonas luteoviolacea* strain S4054, disruption of the *maeA* gene, functionally linked to violacein production, resulted in a beige phenotype. However, pigment production was restored when the mutant was cultivated under highly aerated conditions, suggesting a regulatory influence of oxygen availability on violacein biosynthesis (Thøgersen et al. 2016). This compound exhibits a broad spectrum of biological activities, including antiviral, antibacterial, antioxidant, antiprotozoal, antiparasitary and antitumor effects (Durán et al. 2007, 2012; Balibar and Walsh 2006; Matz et al. 2008). In addition to *P. luteoviolacea*, violacein production has been reported in other purple-pigmented species such as *Pseudoalteromonas amylolytica*, *Pseudoalteromonas umbrosa*, *Pseudoalteromonas ulvae*, and *Pseudoalteromonas byunsanensis*, as well as in the green-pigmented species *Pseudoalteromonas tunicata* (Wu et al. 2017; Thomas et al. 2025; Ayé et al. 2015; Franks et al. 2005; Ramesh et al. 2021). Consistently, our genomic analyses identified the presence of the violacein BGC in all strains exhibiting purple or green pigmentation, further supporting the association between pigment production and the presence of this biosynthetic pathway.

In *Pseudoalteromonas*, yellow and orange pigmentation were not associated with lineage-specific or uniquely conserved biosynthetic gene clusters (BGCs),, indicating the absence of a single genetic determinant underlying these phenotypes. No exclusive BGCs were consistently identified for either coloration. However, BGCs related to tambjamine and arylpolyene-derived biosynthesis were detected among pigmented strains and may contribute to these phenotypes (Franks et al. 2005; Ren et al. 2024). Notably, arylpolyene-related clusters were also widely distributed across both pigmented and non-pigmented strains, indicating that their presence alone is not sufficient to explain pigmentation. This suggests that additional factors, such as gene regulation, pathway interactions, or structural modifications of the final metabolites, likely play a role in determining the observed coloration. The orange phenotype, in particular, may result from the combined activity of multiple pigment-associated pathways or from structural variation in the resulting compounds.

This knowledge gap is further highlighted by the absence of BGCs potentially associated with pigmentation in several yellow and orange-pigmented lineages, including *P. galatheae* and *P. marina*, as well as *P. distincta* and *P. ostreae*. The lack of identifiable pigment-associated BGCs in these strains reinforces the hypothesis that yellow and orange pigmentation may arise from alternative mechanisms, such as non-canonical biosynthetic pathways, pathway interactions, or regulatory processes not captured by current genome mining approaches.

The green pigmentation observed in *P. tunicata* may be explained by the co-occurrence of BGCs associated with prodiginine and tambjamine production. Both compound classes are well-characterized pigmented secondary metabolites in this species, and their combined presence may contribute to the resulting green phenotype, potentially through additive or interactive effects of their respective chromophores.

Brown pigmentation in *Pseudoalteromonas* is likely associated with melanin-like compounds, which are widely reported in marine bacteria and are often linked to stress response mechanisms. Notably, the genetic potential for melanin production appears to be broadly distributed across lineages, including non-pigmented strains, suggesting that its expression is condition-dependent rather than constitutive. In this context, melanin biosynthesis is typically regulated at the transcriptional level and can be induced under environmental stress conditions, such as oxidative stress or nutrient limitation, which may explain its variable phenotypic manifestation (Zeng et al. 2017). This observation further supports the notion that not all pigmentation phenotypes in *Pseudoalteromonas* are driven by specialized metabolism and that some may be linked to more conserved physiological traits. For example, a non-pigmented deep-sea *Pseudoalteromonas* strain (SM9913) was reported to produce a brown pyomelanin-like pigment only under elevated temperature or biofilm conditions, mediated by the induction of the *melA* gene involved in tyrosine catabolism (Zeng et al. 2017). This supports the idea that brown pigmentation may reflect regulated physiological responses rather than constitutive biosynthesis encoded by lineage-specific.

### Core Genome Conservation, Accessory and Singleton Genes in Environmental Adaptation

The intraspecific diversity observed in *Pseudoalteromonas* can result from multiple evolutionary mechanisms, including random homologous recombination, point mutations, multiple essential genes, and the acquisition of niche-adaptive genes, often mediated by horizontal transfer (Balabanova et al. 2023; Okie et al. 2020). These processes confer plasticity to populations, allowing different lineages to coexist or diverge depending on environmental conditions, and favor bacterial speciation through genetic isolation and barriers to recombination. In marine environments, ancestral panmictic populations can rapidly fragment into newly speciated lineages over timescales of tens of thousands of years, primarily when associated with symbionts or subjected to local isolation. These lineages exhibit strong genetic isolation, phylogenetic incongruence among genes, and rearrangements within gene clusters, reflecting ongoing speciation (Wang et al. 2022).

The conservation of metabolic mechanisms in core genes demonstrated in this study, can relatively enable survival and adaptation across diverse marine environments, from sediments to deep and surface waters, and in symbiosis with other organisms. The genus can utilize diverse nutrient sources for survival, demonstrating its resilience, particularly in varied and often oligotrophic marine environments (Holmström and Kjelleberg 1999).

The distribution of COG categories within the *Pseudoalteromonas* core genome is highly similar to that reported for the *Cobetia* core genome (Nedashkovskaya et al. 2024). However, *Pseudoalteromonas* exhibits a higher proportion of genes related to translation, ribosomal structure, and biogenesis, representing approximately 5% more than observed in *Cobetia*, with 15% of conservation. This variety of gene families specialized for translation enables fast and efficient gene expression, consistent with their roles as opportunistic surface colonizers and in symbiotic interactions. Among these genes, we can highlight *groEL*,* groES*,* dnaK*,* dnaJ*,* and tig (Trigger factor)*, which act as chaperones that support protein folding (Tosco et al. 2003; Zhao et al. 2007; Piette et al. 2010). Their conservation in the core genome suggests a fundamental role in maintaining cellular function under diverse environmental conditions. In particular, *groEL* and *groES* act as chaperones with a melting temperature 6 °C lower than that of the homologues in *Escherichia coli*, an adaptation that does not increase catalytic efficiency at low temperatures. However, they present their response as a heat-shock response, indicating that their adaptation is more closely correlated with gene-expression levels and is essential in cold environments (Tosco et al. 2003). Already, the *dnaK* and *dnaJ* genes have been characterized as chaperones involved in adaptation to a psychrophilic lifestyle in *Pseudoalteromonas* sp. SM9913 (Zhao et al. 2007). Interestingly, the importance of *tig* (Trigger factor) is described in *Pseudoalteromonas haloplanktis* TAC125 as the first chaperone to interact with new polypeptides during synthesis in the ribosome, indicating the importance of correct folding at extremely low temperatures in protein synthesis, since the other chaperone genes (such as *dna* and *gro*) are down-regulated at temperatures below 4° C (Piette et al. 2010). This conservation points to a mechanism of protein homeostasis and response to environmental stress when exposed to the marine environment.

Although the core genome reflects a conserved functional backbone across the genus, patterns of genomic diversification are more clearly revealed especially through singleton genes that represent the most lineage-specific fraction of genomic content. Notably, *P. denitrificans* exhibits the highest number of singleton genes among the analyzed genomes, with approximately 2,200 singletons, substantially exceeding the values observed in *P. phenolica* (822), *P. xiamenensis* (789), and *P. obscura* (784), all of which are pigmented strains. *P. denitrificans* also retains accessory non-singleton genes that are more closely related to those found in other species, yet its elevated singleton content contributes to its strong separation in ANI-based analyses. Interestingly, no additional isolates of *P. denitrificans* have been reported in the scientific literature, suggesting that it may represent a relatively underexplored lineage within the genus. These patterns suggest that species differentiation within *Pseudoalteromonas* is not primarily driven by changes in core functional categories, but rather by sequence divergence associated with lineage isolation, leading to genomic separation while preserving functional equivalence. Consistent with this, *P. denitrificans* was originally isolated from seawater collected at 90–100 m depth in a Norwegian fjord system (Enger et al. 1987), an environment that may have contributed to its genomic distinctiveness. Moreover, it possesses a relatively large genome (> 6 Mb) and produces autotoxic compounds that inhibit growth in dense cultures, potentially influencing population structure and ecological interactions. Interestingly, despite producing red pigment, the species is susceptible to UV irradiation, a trait consistent with its deep-sea origin where ultraviolet exposure is limited. Together, these observations indicate that genome diversification in *Pseudoalteromonas* is shaped by a combination of conserved functional stability and lineage-specific gene content, with ecological isolation and restricted gene flow playing key roles in shaping pangenome structure and promoting diversification in marine environments.

### Decoupling Between Secondary Metabolism and Carbohydrate Utilization

In contrast, the relationship between biosynthetic potential and CAZyme content appears to be more limited. Although a weak positive correlation was observed, the low correlation coefficient indicates that secondary metabolism and carbohydrate degradation capacities are only partially linked. This pattern is consistent with our initial hypothesis that CAZyme repertoires are primarily shaped by resource acquisition strategies, whereas BGC diversity reflects chemically mediated ecological interactions such as competition and signaling. Together, these findings reinforce the idea that pigmentation can serve as a proxy for enhanced secondary metabolic potential in *Pseudoalteromonas*, whereas carbohydrate utilization capabilities vary more independently across lineages. This decoupling suggests that different functional traits are subject to separate evolutionary constraints, even within the same genus.

The conservation of these GT families in the core genome suggests a central role in the biosynthesis of surface glycans and exopolysaccharides, as indicated by CAZyme annotations. These molecules are essential for survival under different environmental and temperature conditions, contribute to adhesion to biotic and abiotic surfaces, and support cell envelope integrity. Beyond their ecological importance, the polysaccharide produced by this genus has been studied and applied in the cosmetic industry in recent years, with products for hydration such as the commercial products SeaCode^®^ by Lipotec (Barcelona, Spain) and RefirMAR^®^ by BIOALVO (Lisbon, Portugal) (Rotter et al. 2024; Martins et al. 2014).

*P. carrageenovora* showed in its singletons the presence of carrageenan that targets the β-(1,4) linkages of the λ-carrageenan structure. The ability to degrade λ-carrageenan represents an essential strategy for energy acquisition, since this polysaccharide is highly sulfated and abundant in red algae. Other strains of the genus rely on more than 15 enzymes to achieve a similar metabolic outcome (Vickers et al. 2025). Therefore, the carrageenase produced by *P. carrageenovora* holds significant biotechnological potential, including applications in food processing and biofuel production (Guibet et al. 2007).

The family of PL40, PL25, and PL15 was described only in *P. mariniglutinosa*. The presence of these homologs has been described by ulvan lyase activity, the polysaccharide ulvan is characterized by constituting about 40% of the composition of the green alga Ulvan (Tang et al. 2021). Ulvan lyases degrade this polymer through a β-elimination mechanism that cleaves glycosidic linkages between sulfated rhamnose and uronic acids (glucuronic or iduronic acid), generating unsaturated ulvan-derived oligosaccharides. These oligosaccharides have attracted attention due to their reported biological activities, including antioxidant, antiviral, antibacterial, and antilipidemic properties.

### Phylogenomic Structure and Genome Evolution

Sampling location did not significantly influence BGC abundance or CAZyme profiles, suggesting that these traits are more strongly associated with phylogenetic background or microenvironmental conditions than with geographic origin. Although a weak positive correlation was observed between biosynthetic potential and CAZyme content, the low coefficient indicates only partial linkage between secondary metabolism and carbohydrate degradation. Non-pigmented lineages did not show increased CAZyme abundance; however, literature reports indicate that these strains harbor specialized enzymes (e.g., carrageenases, chitinases, alginate lyases) and exhibit broader environmental tolerance and metabolic versatility (Bowman 2007). Thus, CAZyme repertoires in these lineages are likely functionally optimized rather than expanded, reflecting adaptation to variable resource availability, whereas BGC diversity is more related to ecological interactions and competition.

Consistent with this framework, our results showed a clear genomic compartmentalization of pangenome fractions across replicons. Core gene families were significantly enriched in the primary chromosome, whereas accessory gene families were significantly enriched in secondary chromosomes. Singleton genes displayed a weaker but still significant enrichment in secondary chromosomes and showed the highest variability among genomes, indicating that recently acquired genes are more dynamically distributed across multipartite genomes. The multipartite genome architecture of *Pseudoalteromonas*, particularly the presence of a secondary chromosome enriched in accessory genes, provides a mechanistic basis for extensive gene gain and adaptation (Sonnenberg and Haugen 2021). Consistent with this framework, our results show a clear genomic compartmentalization of pangenome components across replicons, with core gene families predominantly concentrated in the primary chromosome, whereas accessory genes are significantly enriched in secondary replicons (Fig. S2). This organization supports the hypothesis that secondary chromosome function as specialized reservoirs for accessory functions, reinforcing genome plasticity and facilitating lineage-specific adaptation.

Recent analyses have further demonstrated that bipartite genomes, including *Pseudoalteromonas*, exhibit higher pangenome openness compared to monopartite bacteria, with shell and cloud gene categories driving most of this expansion (Sonnenberg and Haugen 2023). In agreement with this pattern, singleton genes in our dataset displayed variability and a tendency to accumulate in smaller replicons, particularly in third replicons, suggesting that the most recently acquired genes are not randomly distributed but instead partially structured by genomic architecture. Together, these observations support the idea that secondary chromosome contribute directly to the dynamic gene gain observed across lineages and reinforce the open nature of the *Pseudoalteromonas* pangenome.

The elevated pangenome openness observed in bipartite genomes reflects an increased propensity for gene acquisition and genomic innovation (Medini et al. 2005; Sonnenberg and Haugen 2023). In our dataset, gene content reconstruction further supports this expansion-driven model, with ancestral genome estimates (~ 2,999 genes) substantially lower than those observed in extant lineages (~ 4,245 genes), indicating genome expansion primarily driven by gene gain (Sonnenberg and Haugen 2021). The predominance of gene acquisition in terminal branches highlights the dynamic nature of the accessory genome, which underpins niche differentiation and ecological performance, consistent with previous studies (Parrilli et al. 2019; Bosi et al. 2017).

This pattern aligns with the open pangenome structure observed here (α = 0.57), characterized by a small conserved core and a large accessory fraction. Similar to other marine genera such as *Shewanella* pangenome has been described as equally open, containing 13,406 non-redundant genes and a core of 1,878 genes across 24 *Shewanella* genomes (Zhong et al. 2018). Likewise, the genus *Aliivibrio* comprises 9,469 gene clusters, of which only 1,857 belong to the core genome, while 7,612 constitute the accessory fraction and as *Alteromonas* (1,795 core genes and 7,828 flexible genes) (Scherer et al. 2026; López-Pérez and Rodriguez-Valera 2016). The core genome functional profile is also consistent with that of *Cobetia* (Nedashkovskaya et al. 2024), although the higher proportion of translation-related genes and the elevated singleton content in *P. denitrificans* suggest enhanced adaptive capacity and lineage-specific divergence. Overall, the observed intraspecific diversity reflects well-established evolutionary mechanisms, including recombination, mutation, and horizontal gene transfer (Balabanova et al. 2023; Okie et al. 2020), which promote rapid lineage differentiation and genetic isolation in marine environments (Wang et al. 2022). The broad ecological distribution of *Pseudoalteromonas*, spanning pelagic habitats, sediments, and associations with diverse marine organisms, likely increases opportunities for horizontal gene transfer and contributes to the maintenance of an expansive accessory genome (Zhong et al. 2018). Together, these findings support a model in which a conserved functional backbone coexists with a highly flexible accessory genome, driving ecological adaptation, genomic innovation, and diversification within the genus.

### Implications for Bioprospecting

Together, our results address key challenges in genus-level pangenomic analyses of *Pseudoalteromonas*, particularly the accurate inference of core and accessory gene sets across diverse genomes. By applying an integrative framework that overcomes limitations associated with sequence similarity thresholds and scaling of conventional approaches, this study provides a consistent view of pangenome structure at the genus level. The results confirm the open nature of the pangenome and the presence of a conserved core genome alongside a large and variable accessory fraction. While the core genome supports essential cellular functions, the accessory and singleton gene pools account for most of the observed variation and are associated with metabolic diversification. The absence of consistent associations with pigmentation or geographic origin indicates that these traits are not reliable predictors of functional potential. In this context, the high proportion of poorly characterized genes and biosynthetic clusters reinforces the importance of pangenomic approaches for guiding bioprospecting strategies. The enrichment of biosynthetic potential in specific lineages, particularly pigmented strains, supports the use of targeted approaches for the discovery of novel compounds. In addition, the dynamic nature of the accessory genome suggests that recently diversified lineages represent a relevant source of new secondary metabolites. Overall, these findings demonstrate that linking pangenome structure to metabolic potential provides a practical framework to prioritize strains and improve the efficiency of bioprospecting in *Pseudoalteromonas*.

## Supplementary Information

Below is the link to the electronic supplementary material.

Supplementary Material 1 (DOCX 2.74 MB)

## Data Availability

All data supporting the findings of this study are available within the paper and its Supplementary Information.
