# Supplementary material for: Pangenome Dynamics and Functional Diversification in the Marine Genus Pseudoalteromonas: Association to Colony Pigmentation
Source: Mar Biotechnol (NY). 2026 Jul 20;28(4):122. doi: 10.1007/s10126-026-10674-7 (PMC13385065; doi:10.1007/s10126-026-10674-7)
Supplement: Supplementary file 1 — Supplementary Material 1 (DOCX 2.74 MB) [file 10126_2026_10674_MOESM1_ESM.docx]

**Pangenome dynamics and functional diversification in the marine genus *Pseudoalteromonas*: association to colony pigmentation**

Jéssica Scherer¹ ², Renato Kulakowski Corá², Diego Bonatto³, Alexandre José Macedo¹ ² *

¹ Laboratório de Biofilmes e Diversidade Microbiana, Faculdade de Farmácia e Centro de Biotecnologia, Universidade Federal do Rio Grande do Sul, Porto Alegre 91501-970, Rio Grande do Sul, Brazil.

² Regenera Moléculas do Mar – Avenida Ipiranga 6681, prédio 96D, sala 210 – CEP 90160-091, Partenon, Porto Alegre, RS, Brazil.

³ Laboratório de Biologia Molecular e Computacional, Centro de Biotecnologia da UFRGS, Departamento de Biologia Molecular e Biotecnologia, Universidade Federal do Rio Grande do Sul, Porto Alegre, RS, Brazil.

*Correspondence: alexandre.macedo@ufrgs.br

**Supplementary materials**

Table S1. The accession numbers and general attributes of the *Pseudoalteromonas* spp. genomes used in pangenome.

| **Species as originally identified** | **Strain** | **Accession Genome** | **Size (b)** | **G + C (mol%)** | **Completeness (%)** | **Contamination (%)** | **Isolation source** | **Colour** |
| --- | --- | --- | --- | --- | --- | --- | --- | --- |
| *Pseudoalteromonas xiamenensis* | CGMCC 1.12157 | GCF_042431545.1-RS_2024_09_29 | 4622515 | 43 | 99.7 | 1 | Seawater | Red |
| *Pseudoalteromonas viridis* | BBR56 | GCF_017742995.1-RS_2025_04_28 | 5549631 | 49.5 | 98.95 | 0.51 | Seawater | Red |
| *Pseudoalteromonas undina* | DSM 6065 | GCF_000238275.3-RS_2025_03_28 | 4027610 | 40 | 99.82 | 0.25 | Seawater | Non-pigmented |
| *Pseudoalteromonas umbrosa* | B95 | GCF_030125375.1-RS_2025_03_02 | 6189459 | 41.5 | 99.95 | 2.63 | Montipora capitata Coral | Brown |
| *Pseudoalteromonas ulvae* | TC14 | GCF_002156545.1-RS_2025_04_27 | 4586484 | 41.5 | 99.95 | 0.7 | Biofilm Toulon harbor | Purple |
| *Pseudoalteromonas tunicata* | D2 | GCF_003568825.1-RS_2025_03_28 | 4967522 | 40 | 99.7 | 0.13 | Tunicate Ciona intestinalis | Green |
| *Pseudoalteromonas translucida* | TAC125 | GCF_000026085.1-RS_2024_12_13 | 3850272 | 40 | 99.98 | 0.25 | Antarctic coastal sea water | Non-pigmented |
| *Pseudoalteromonas tetraodonis* |  | GCF_046712425.1-RS_2025_01_16 | 3910324 | 40 | 98.18 | 0.51 | Sea ice | Non-pigmented |
| *Pseudoalteromonas spongiae UST010723-006* | UST010723-006 | GCF_000238255.3-RS_2025_03_29 | 4752413 | 41 | 99.94 | 0.38 | Sponge Mycale adhaerens | Orange |
| *Pseudoalteromonas simplex* | A520 | GCF_015278155.1-RS_2024_12_09 | 4501926 | 41 | 99.95 | 0.25 | Sphoeroides spengleri | Yellow |
| *Pseudoalteromonas shioyasakiensis* | JCM 18891 | GCF_001550135.1-RS_2025_04_22 | 4807552 | 41.5 | 99.95 | 01.07 | Sediment ocean | Non-pigmented |
| *Pseudoalteromonas ruthenica* | S3137 | GCF_000967625.1-RS_2025_03_28 | 4066635 | 47.5 | 99.49 | 0.72 | Seaweed | Orange |
| *Pseudoalteromonas rubra* | DSM 6842 | GCF_000238295.3-RS_2025_04_06 | 6143755 | 48 | 99.71 | 0.93 | Seawater | Red |
| *Pseudoalteromonas rhizosphaerae* | hCg-42 | GCF_028885455.1-RS_2025_05_12 | 5358982 | 40.5 | 10000% | 4.86 | Magallana gigas hemolymph | Non-pigmented |
| *Pseudoalteromonas qingdaonensis* | YIC-827 | GCF_038739665.1-RS_2025_05_02 | 3265653 | 49 | 97.56 | 0.67 | Intestines of marine benthic organisms | Non described |
| *Pseudoalteromonas prydzensi* | ACAM 620 | GCF_014925355.1-RS_2024_11_20 | 5205557 | 41 | 99.75 | 2.15 | Sea ice | Non-pigmented |
| *Pseudoalteromonas piscicida* | WCPW15003 | GCF_019797925.1-RS_2025_03_30 | 5380608 | 43.5 | 90.15 | 6.55 | Mariculture | Orange |
| *Pseudoalteromonas piratica* | OCN003 | GCF_000788395.1-RS_2024_12_09 | 4815987 | 40 | 100 | 1.44 | Montipora capitata coral fragment | Yellow |
| *Pseudoalteromonas phenolica* | KCTC 12086 | GCF_001444405.1-RS_2025_03_03 | 4868993 | 40.5 | 99.95 | 1.12 | Seawater | Brown |
| *Pseudoalteromonas pernae* | YIC-656 | GCF_039831865.1-RS_2025_08_15 | 3973550 | 45.5 | 99.41 | 0.59 | Intestinal tract of Perna mussels (Bivalvia: Mytilidae) | Non described |
| *Pseudoalteromonas peptidolytica* | NBRC 101021 | GCF_007989895.1-RS_2025_09_09 | 5036479 | 42.5 | 99.49 | 1.22 | Seawater | Yellow |
| *Pseudoalteromonas ostreae* | hOe-124 | GCF_029023665.1-RS_2025_05_12 | 4735317 | 40.5 | 99.12 | 1.26 | Magallana gigas hemolymph | Orange |
| *Pseudoalteromonas obscura* | P94 | GCF_030135505.1-RS_2025_03_02 | 6295190 | 42.5 | 99.7 | 0.41 | Montipora capitata | Purple |
| *Pseudoalteromonas nigrifaciens* | KMM 661 | GCF_002221505.1-RS_2025_04_22 | 4274777 | 40 | 99.92 | 0.54 | Mussels | Brown |
| *Pseudoalteromonas neustonica* | PAMC 28425 | GCF_001653135.1-RS_2025_08_12 | 4972584 | 39.5 | 99.75 | 0.88 | Seawater | Orange |
| *Pseudoalteromonas mariniglutinosa* | NCIMB 1770 | GCF_021613355.1-RS_2025_03_11 | 5031620 | 41 | 99.49 | 1.47 | Diatom seawater | Non-pigmented |
| *Pseudoalteromonas marina* | 13-15 | GCF_900141965.1-RS_2025_04_27 | 4099967 | 40 | 100 | 0.13 | Tidal flat sediment | Yellow |
| *Pseudoalteromonas maricaloris* | SCSIO 43202 | GCF_024746855.1-RS_2025_02_16 | 5416459 | 43.5 | 100 | 0.41 | Galaxea fascicularis | Yellow |
| *Pseudoalteromonas luteoviolacea* | H2 | GCF_006704145.1-RS_2025_04_27 | 6043625 | 42 | 96.42 | 6.78 | Coral reef | Purple |
| *Pseudoalteromonas lipolytica* | HJ51 | GCF_003515105.1-RS_2025_04_22 | 4733916 | 41.5 | 99.95 | 01.01 | Seawater | Non-pigmented |
| *Pseudoalteromonas issachenkonii* | KMM 3549 | GCF_002310795.1-RS_2025_04_22 | 4132618 | 40.5 | 99.95 | 0.51 | Brown alga seawater Fucus evanescens | Non-pigmented |
| *Pseudoalteromonas holothuriae* | CIP 111854 | GCF_945859825.1-RS_2025_03_02 | 5177136 | 40.5 | 99.75 | 0.83 | Wild sea cucumbers | Brown |
| *Pseudoalteromonas haloplanktis* | CIP 103197 | GCF_945859885.1-RS_2025_03_16 | 3972527 | 40 | 86.02 | 4.2 | Seawater | Non-pigmented |
| *Pseudoalteromonas gelatinilytica* | NH153 | GCF_001641615.1-RS_2025_03_28 | 4797622 | 41.5 | 99.95 | 0.65 | Seawater | Non-pigmented |
| *Pseudoalteromonas galatheae* | S4498 | GCF_005886105.2-RS_2024_12_11 | 5366397 | 43 | 99.62 | 0.67 | Shrimp | Yellow |
| *Pseudoalteromonas fuliginea* | KMM 216 | GCF_000690055.1-RS_2025_08_06 | 4766895 | 39 | 99.75 | 0.57 | Bay | Brown |
| *Pseudoalteromonas fenneropenaei* | KCTC 42730 | GCF_042643375.1-RS_2024_10_06 | 4651258 | 45.5 | 99.7 | 0.8 | Sediment of a pond containing farmed Fenneropenaeus | Non-pigmented |
| *Pseudoalteromonas espejian* | DSM 9414 | GCF_002221525.1-RS_2025_05_03 | 4500451 | 40.5 | 99.49 | 0.51 | Seawater | Non-pigmented |
| *Pseudoalteromonas distincta* | 16-SW-7 | GCF_005877035.1-RS_2025_08_18 | 4531445 | 39.5 | 100 | 0.38 | Seawater | Orange |
| *Pseudoalteromonas denitrifican* | DSM 6059 | GCF_900112265.1-RS_2025_05_03 | 6094166 | 34.5 | 97.22 | 3.67 | Fjord system off | Red |
| *Pseudoalteromonas citrea* | DSM 8771 | GCF_000238375.3-RS_2025_03_23 | 5434598 | 41 | 99.75 | 0.13 | Seawater | Yellow |
| *Pseudoalteromonas carrageenovora* | ATCC43555T | GCF_900239935.1-RS_2024_12_13 | 4584980 | 39.5 | 99.21 | 0.25 | Pool of bacteria from marine waters and algae | Non-pigmented |
| *Pseudoalteromonas caenipelagi* | JBTF-M23 | GCF_013140855.1-RS_2025_08_07 | 5082770 | 42 | 99.92 | 0.7 | Tidal flat | Yellow |
| *Pseudoalteromonas byunsanensis* | JCM 12483 | GCF_001854475.1-RS_2025_05_02 | 4743224 | 42.5 | 99.75 | 1.37 | Tidal flat | Purple |
| *Pseudoalteromonas aurantia* | 208 | GCF_014858725.1-RS_2024_11_18 | 5602374 | 41 | 100 | 0.48 | Seawater | Orange |
| *Pseudoalteromonas atlantica* | NBRC 103033 | GCF_007988745.1-RS_2024_11_18 | 4468419 | 41 | 99.24 | 0.25 | Palmaria palmata | Non-pigmented |
| *Pseudoalteromonas ardens* | R96 | GCF_030125445.1-RS_2025_03_02 | 5937012 | 48 | 99.96 | 0.76 | Montipora capitata | Red |
| *Pseudoalteromonas arctica* | A 37-1-2 | GCF_000238395.3-RS_2024_09_03 | 4721971 | 39 | 99.75 | 0.38 | Seawater | Orange |
| *Pseudoalteromonas arabiensis* | JCM 17292 | GCF_001550155.1-RS_2025_03_28 | 4459111 | 41 | 99.95 | 0.43 | Ocean sediment | Non-pigmented |
| *Pseudoalteromonas apostichopi* | FE4 | GCF_030297135.1-RS_2025_06_30 | 4455557 | 41 | 99.95 | 0.13 | fertilized egg Apostichopus japonicus | Non-pigmented |
| *Pseudoalteromonas amylolytica* | JW1 | GCF_001854605.1-RS_2025_03_28 | 4856394 | 43.5 | 99.49 | 0.54 | Seawater | Purple |
| *Pseudoalteromonas aliena* | EH1 | GCF_001999225.1-RS_2025_03_05 | 4594697 | 39 | 100 | 0.66 | Seawater | Brown |
| *Pseudoalteromonas agarivorans* | DK3 | GCF_024518735.1-RS_2025_03_30 | 4465559 | 41 | 100 | 0.57 | Algae | Non-pigmented |


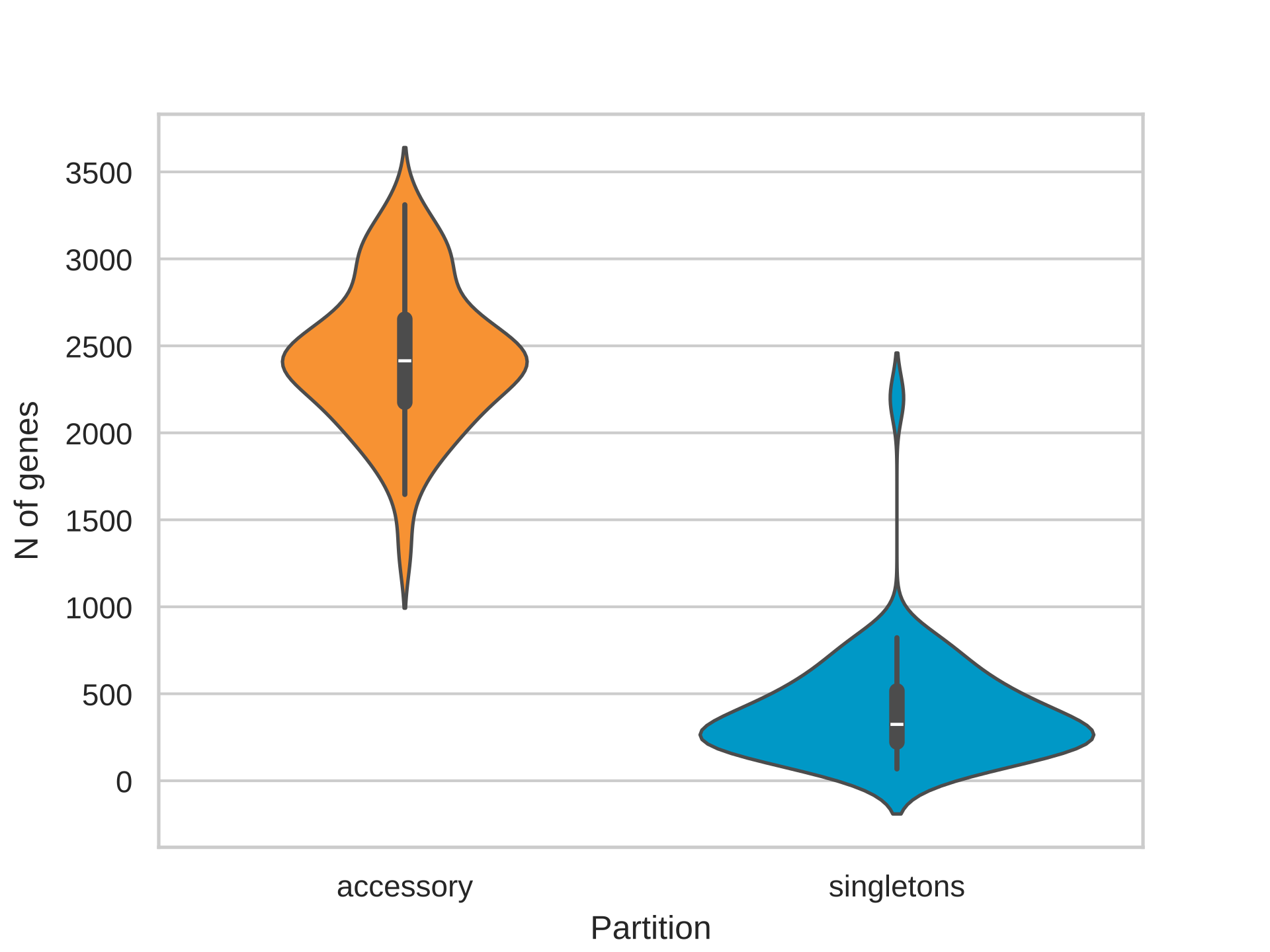


**Fig. S1.** Distribution of accessory and singletons genes in *Pseudoalteromonas* genomes. Violin plots illustrate the distribution and density of accessory (orange) and singleton (blue) gene families across the analyzed genomes. The vertical range indicates variability in gene counts among genomes, whereas the violin width represents the density of observations

a)


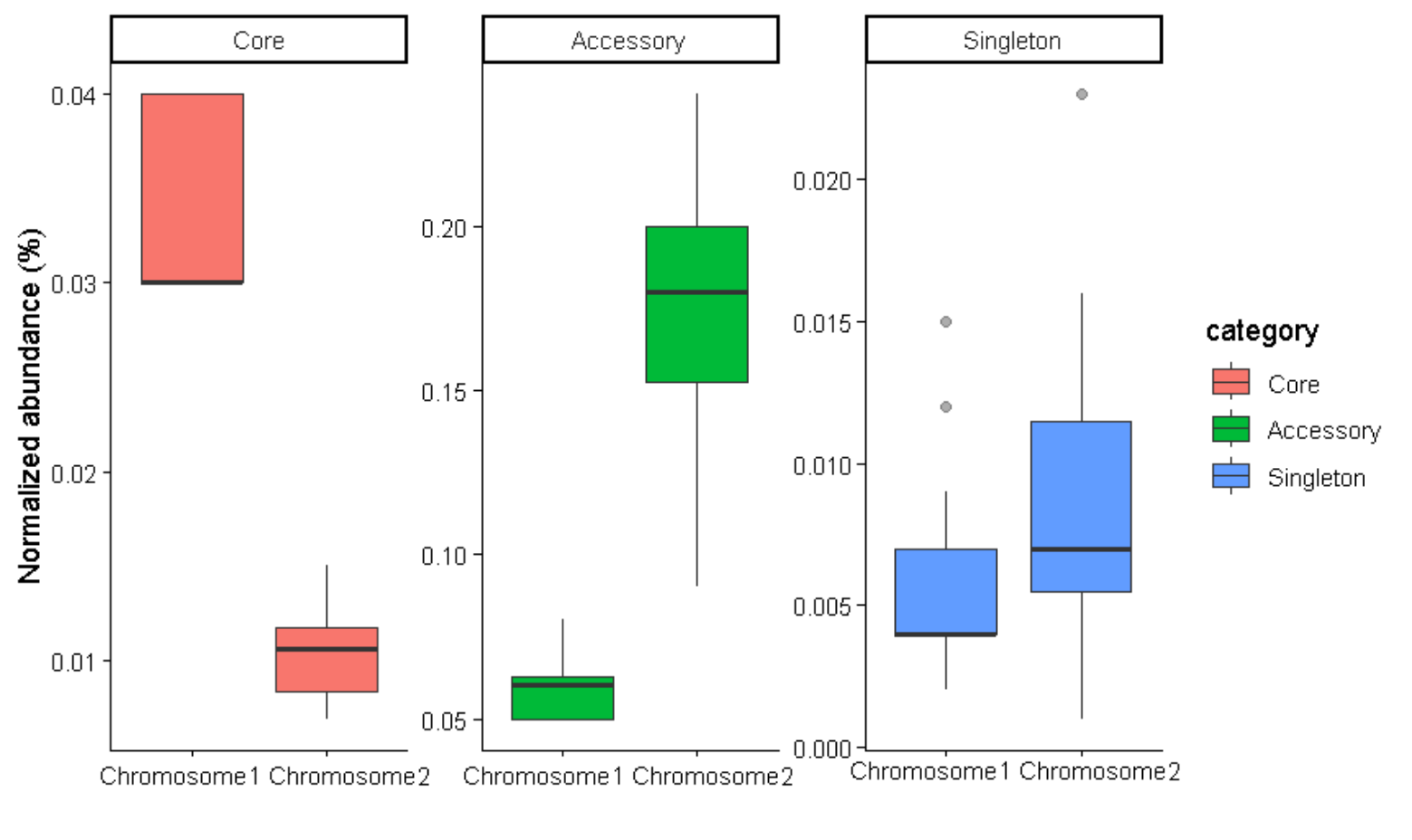


b)


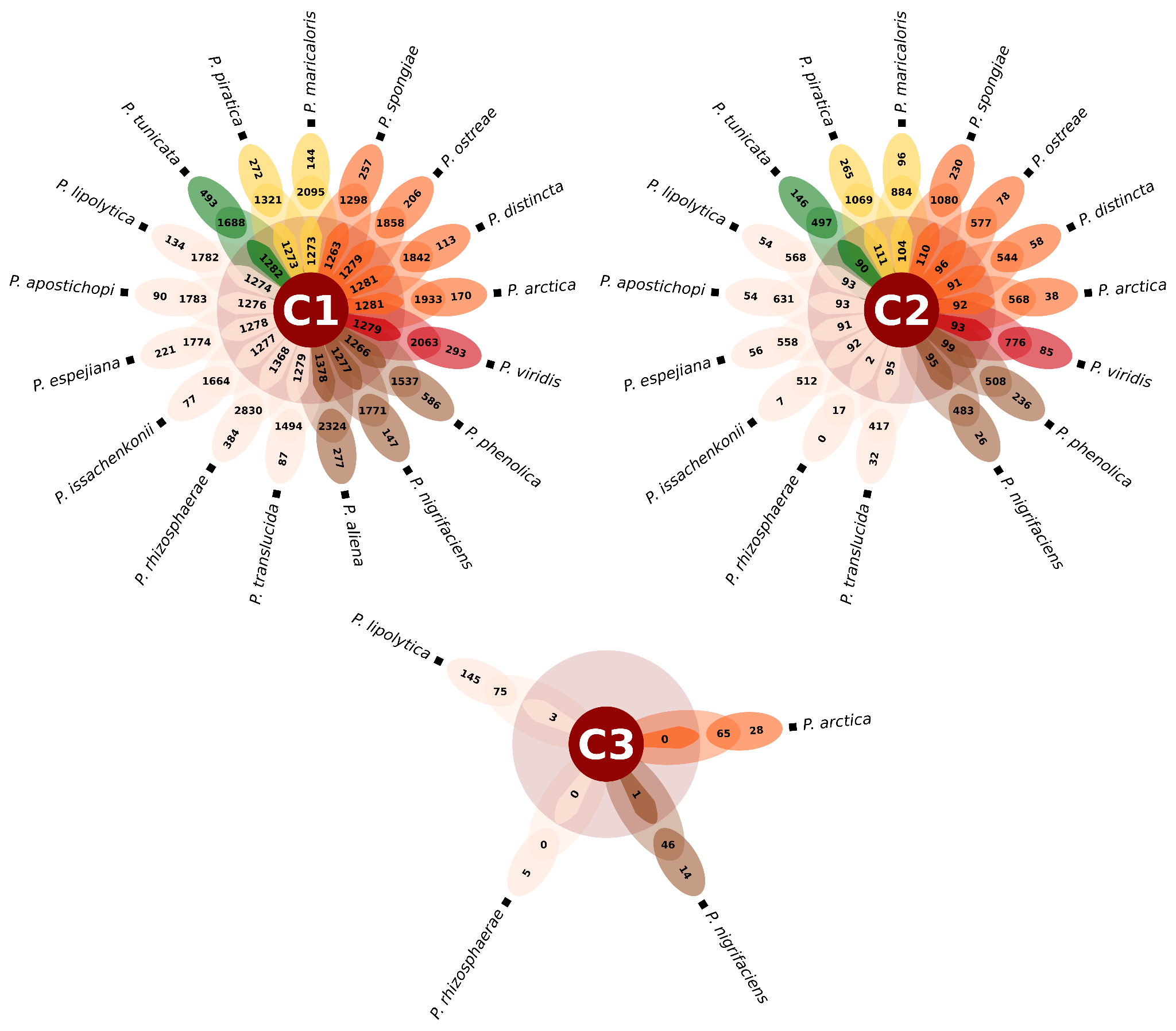


**Fig. S2** Genomic distribution of pangenome *Pseudoalteromonas* spp*.* by categories across replicons of 16 genomes. a) Boxplot normalized abundance (%) of Core, Accessory, and Singleton gene categories evaluated in bacterial Chromosome 1 and chromosome 2. Boxplots display the median (horizontal line), interquartile range (box), and potential outliers (dots), with individual vertical axes scaled freely to optimize category visualization. b) Distribution of genes in the genomes of *Pseudoalteromonas* spp.by flower plots showing the core gene number (in the center), accessory gene number (in the petals), and strain-specific gene number (at the tip of the petals). C1: Chromosome 1, C2: Chromosome 2, C3: Plasmide.


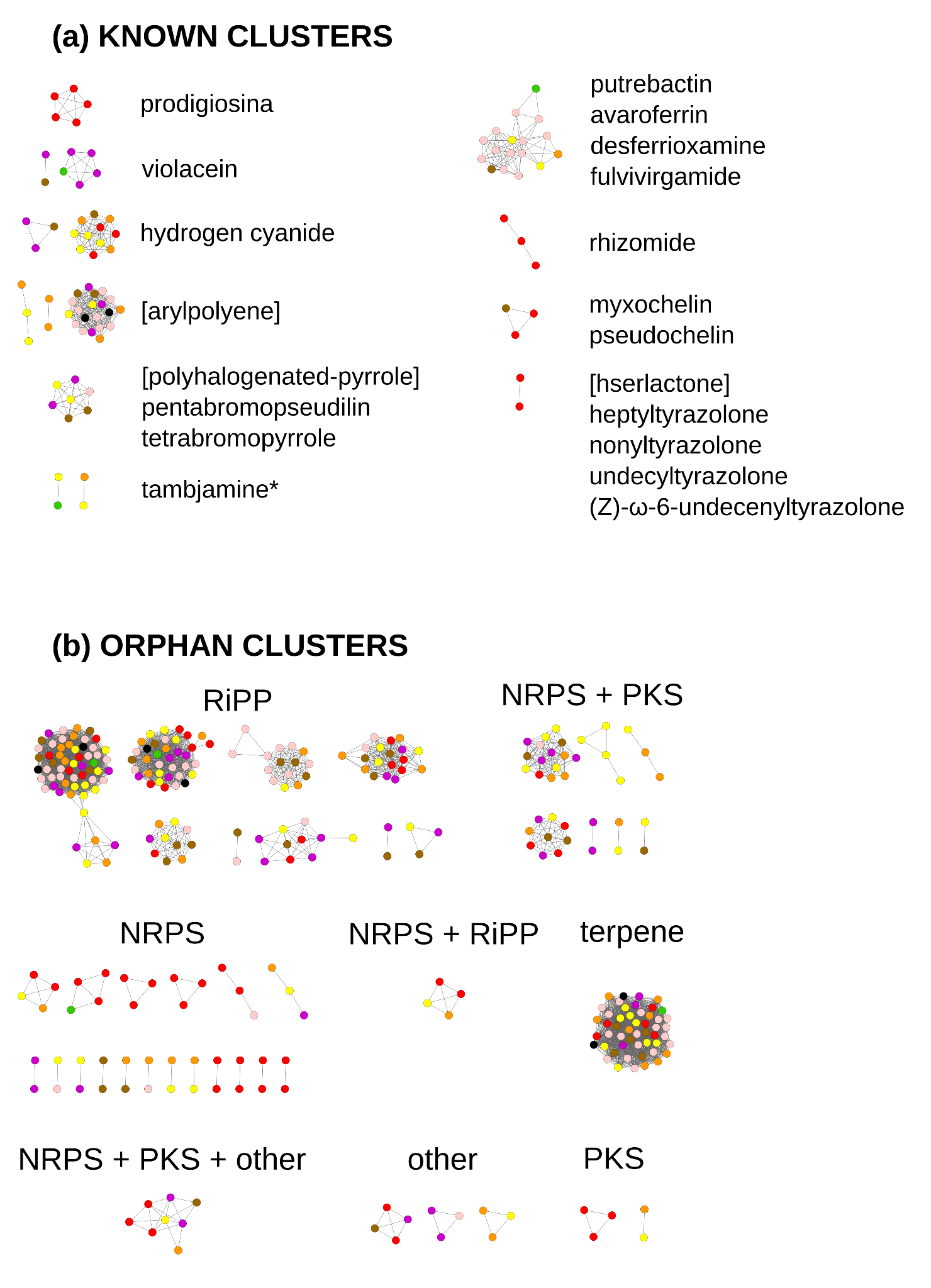


**Fig. S3** Global network view of biosynthetic gene clusters BGCs across *Pseudoalteromonas* genomes, grouped into major biosynthetic families. Each node represents a biosynthetic gene cluster (BGC) with node color indicating the pigmentation of the corresponding strain and edges indicate similarity relationships identified by BIG-SCAPE. A) Non-Ribosomal Peptide Synthetase (RiPPs). B) Ribosomally synthesized and Post-translationally modified Peptides (NRPS). C) Polyketide Synthase (PKS). D) Others. E) Terpene. Only biosynthetic gene clusters classified with high confidence were considered for analysis, with the exception of the indication (*) cluster, which was included based on manual curation following in-depth cluster analysis


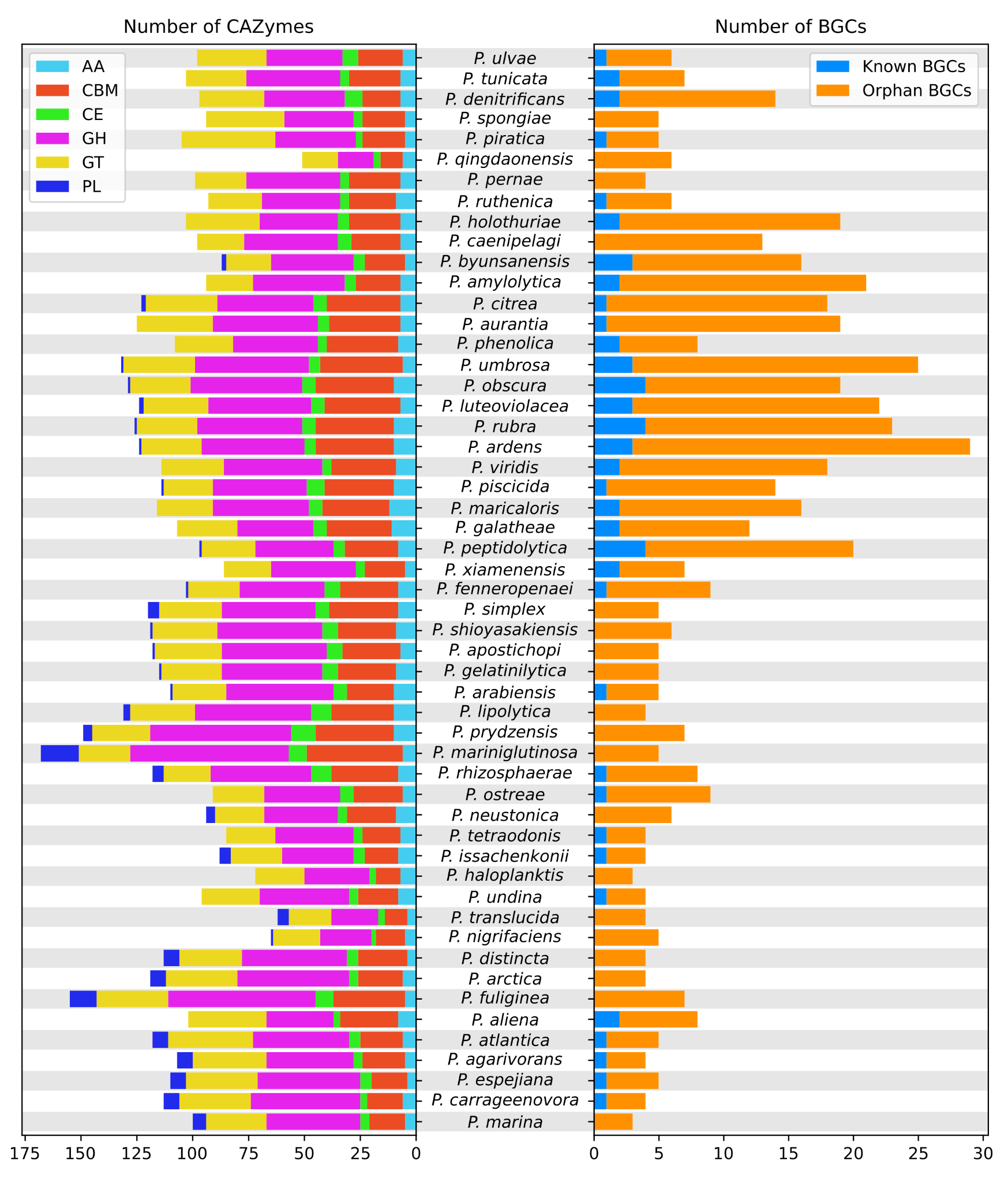


**Fig. S4** Species-level comparison of functional repertoires in *Pseudoalteromonas*. For each species, the total number of carbohydrate-active enzymes (CAZymes) is shown on the left, while the number of biosynthetic gene clusters (BGCs) predicted by antiSMASH is shown on the right. Species names are positioned centrally to facilitate direct comparison. The proportion of known and orphans BGCs are indicated by color, as well as the CAZyme families. AA: Auxiliary Activities; CBM: Carbohydrate-Binding Modules; CE: Carbohydrate Esterases; GH: Glycoside Hydrolases; GT: GlycosylTransferases; PL: Polysaccharide Lyases


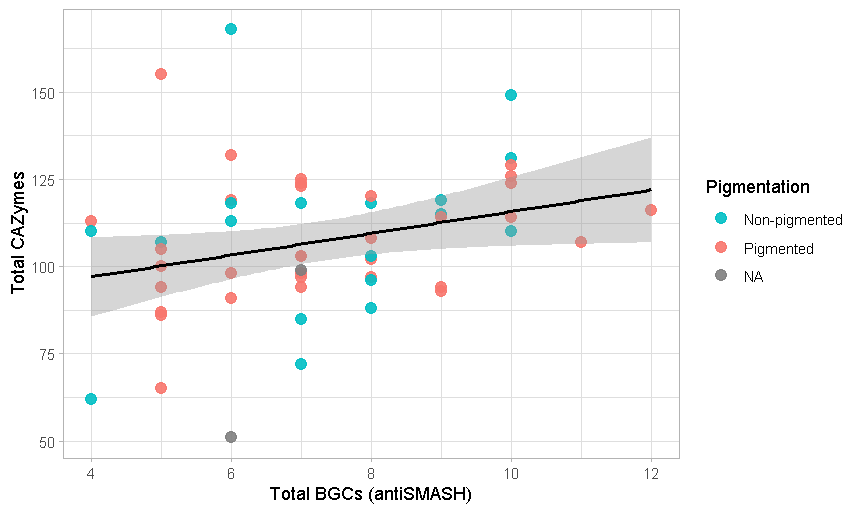


**Fig. S5** Relationship between biosynthetic gene clusters (BGCs) and carbohydrate-active enzymes (CAZymes) in *Pseudoalteromonas* by pigmentation. The scatter plot shows the total number of antiSMASH-predicted BGCs versus CAZyme counts for each genome

1. **
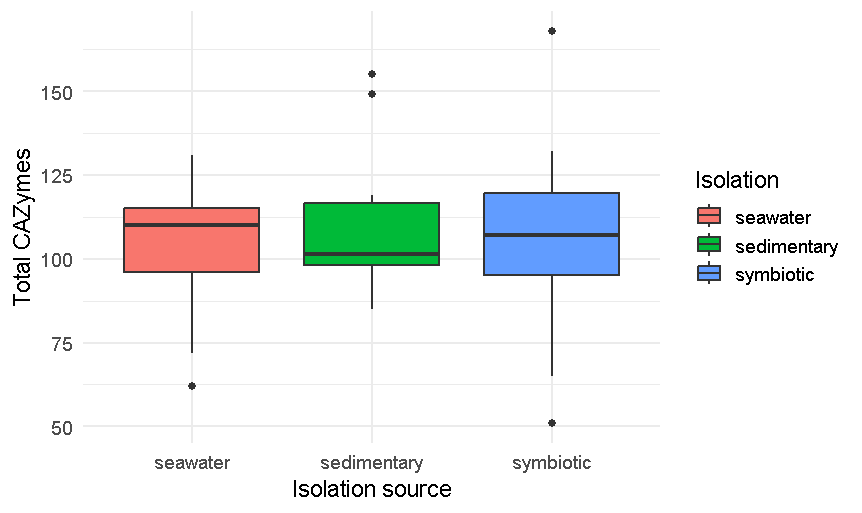
**

**
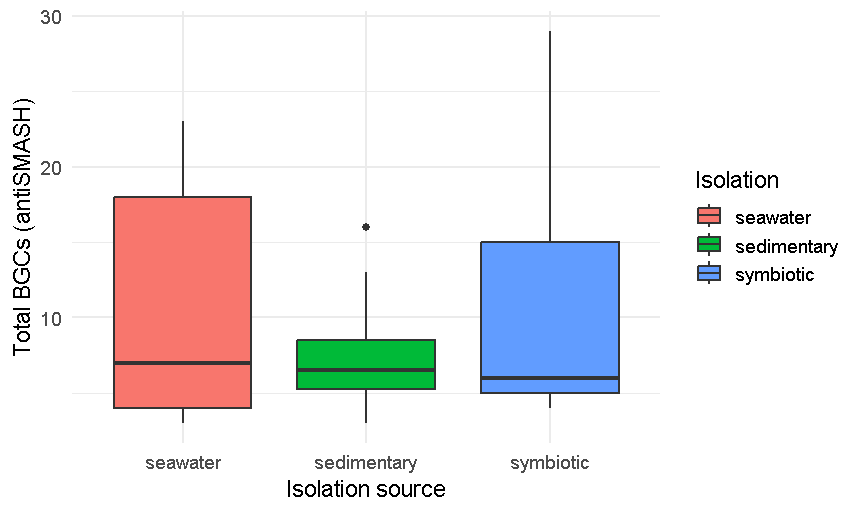
**

**Fig. S6** Relationship between isolation source and a) CAZYmes data and b) biosynthetic gene clusters (BGCs)
